# The causal effect of oxidative stress on the risk of keratoconus: A bidirectional two-sample Mendelian randomization study

**DOI:** 10.1097/MD.0000000000046055

**Published:** 2025-12-19

**Authors:** Jiao Wang, Jie Zhang, Qianqian Sun

**Affiliations:** a Department of Clinical Laboratory, Beijing Anzhen Nanchong Hospital of Capital Medical University & Nanchong Central Hospital, Nanchong, China; b Reproductive Medicine Department, Affiliated Hospital of North Sichuan Medical College, Nanchong, China; c Department of Clinical Laboratory, Zhengzhou Key Laboratory of Children’s Infection and Immunity, Children’s Hospital Affiliated to Zhengzhou University, Zhengzhou, China.

**Keywords:** keratoconus, Mendelian randomization study, oxidative stress

## Abstract

Keratoconus (KC) is an intricate disease involving multiple factors. It is widely accepted that the pathogenesis of KC is linked to oxidative stress (OS). Nevertheless, the precise causal relationship between OS and KC remains uncertain due to the presence of confounding factors. This study aims to ascertain whether there was a potential causal effect between OS and KC. A bidirectional 2-sample Mendelian randomization (MR) analysis was conducted, utilizing genetic instrumental variables as substitutes for 12 oxidative stress injury biomarkers (OSIBs). The available summarized data for OSIBs were obtained through the published genome-wide association study. Data for KC was collected from the FinnGen cohort, comprising 311 cases and 209,287 controls of European population. The primary MR analysis employed the inverse variance weighted (IVW) method. To evaluate the reliability of the observed associations, sensitivity analysis and reverse MR analysis were performed. The MR analysis revealed significant associations. The IVW method suggested that elevated genetically predicted glutathione peroxidase levels were correlated with the diminished KC risk (odds ratio [OR]: 0.660, 95% confidence interval [CI]: 0.500–0.873, *P* = .004). Total bilirubin was also found to be associated with a decreased risk of KC through the IVW method (OR: 0.912, 95% CI: 0.845–0.984, *P* = .017), which was consistent with the result from the MR-Egger method (OR: 0.910, 95% CI: 0.835–0.992, *P* = .034). Reverse MR analysis did not suggest causal relationship of KC on OSIBs. These findings provide a robust support for the causal link between OSIBs and the development of KC, indicating that targeting OS pathways may become a potential therapeutic approach for KC.

## 1. Introduction

Keratoconus (KC) is a progressive and asymmetrical corneal ectasia characterized by central corneal thinning and conical protrusion. The condition typically results in irregular astigmatism, irreversible vision loss, corneal scarring and other visual impairments, seriously impacting the quality of life for those affected.^[1]^ The disease manifests predominantly in males during their late teens and in females during their early twenties. By around the age of 40, this process frequently stabilizes.^[2]^ It is estimated that the occurrence rate varies between 1.5 and 25 instances per 100,000 individuals annually. There is a higher occurrence of this condition among individuals residing in the Middle East and Asia, as evidenced by research findings.^[3]^ Therefore, it is urgent to identify the key influencing factors of KC, which is crucial for reducing the high prevalence of KC.

KC is a complex multifactorial disease, influenced by multiple factors, divided broadly into genetic and environmental categories.^[4]^ It is noteworthy that oxidative stress (OS) appears to be the convergent pathway through which these disparate factors exert their pathological effects,^[5]^ and is also regarded as an important factor for several ocular diseases, included KC.^[6,7]^ Numerous studies have suggested that oxidative stress injury biomarkers (OSIBs) are disordered in the cornea or different body fluids of KC.^[8]^ Observational research indicates that the level of catalase (CAT) activity in the cornea is reduced in patients with KC compared to individuals without the condition. The reduction in activities is associated with higher KC stages.^[9]^ A diminution in the activity of superoxide dismutase (SOD) and glutathione has been detected in the blood of individuals diagnosed with KC, accompanied by increased levels of CAT.^[10]^ As a nonenzymatic molecule antioxidant, uric acid (UA) is found to be elevated in the tear film of KC patients, implying its possibility as a risk factor.^[11]^ Interestingly, vitamin C levels appear to rise in KC corneas,^[12]^ indicating an adaptive reaction to OS and compromised collagen production. These findings suggest a strong link between OSIBs and the pathogenesis of KC. Nevertheless, there is presently inadequate proof to demonstrate the particular causal connection between these elements, chiefly owing to the impact of confounding elements and reverse causality in observational studies.

Mendelian randomization (MR) is an instrumental variable-based approach to analyze causal connections between exposures and outcomes.^[13,14]^ In contrast to conventional epidemiological investigations, the MR study has the capability to address underlying confounding variables and eliminate reverse causality by employing genetic variants as instrumental variables (IVs), which is widely used for investigating causal effects.^[15]^

This study seeks to explore the causal link between OSIBs and KC through the utilization of the bidirectional 2-sample MR method. By employing extensive genome-wide association studies (GWAS) datasets of OSIBs and KC, we can surmount the constraints of observational studies and derive more reliable causal judgments. This bidirectional analysis will enable us to investigate the impact of OSIBs on the development of KC and explore the possibility of a reverse causal relationship.

## 2. Materials and methods

### 2.1. Study design

**Figure 1.**
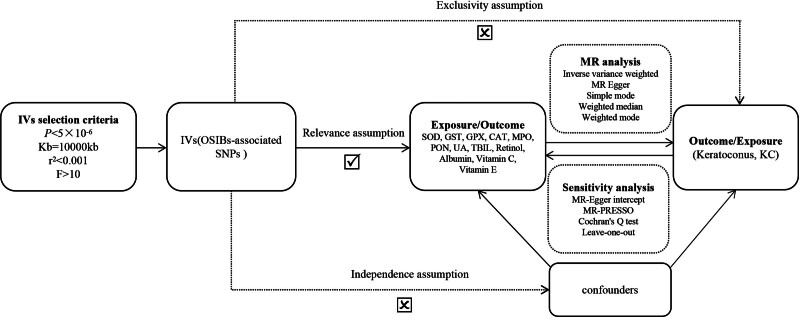
The flowchart of the study design in the bidirectional MR analysis. MR = Mendelian randomization.

A bidirectional MR design was employed to investigate the causal relationship between OSIBs and the risk of KC in this research. Genetic variants linked to exposures (OSIBs) were identified as IVs and were subsequently evaluated for their combined impact on the outcome (KC). Our MR analysis followed these 3 basic assumptions: Relevance: there was a strong correlation between IV and exposure. Independence: IV was independent of any confounding factors affecting the exposure–outcome relationship. Exclusivity: IV only affected the results through its impact on exposure. This research was performed strictly in accordance with the STROBE-MR guidelines.^[16]^ The MR design flowchart of this study is shown in Figure 1. Reverse MR analysis was applied to avoid possible reverse effects. Since the data originated from publicly accessible GWAS, there was no necessity to obtain ethical approval from a committee.

### 2.2. Data sources

We retrieved the genetic data of KC and OSIBs from the IEU OpenGWAS project. 12 biomarkers of OS were included, specifically, SOD, glutathione S-transferase (GST), glutathione peroxidase (GPX), CAT, myeloperoxidase (MPO), paraoxonase (PON), UA, total bilirubin (TBIL), retinol, albumin, vitamin C, and vitamin E. Summary data of KC were sourced from the FinnGen consortium (GWAS ID: finn-b-H7_CORNEALDEFORM) including 311 cases and 209,287 controls. All individuals who took part in these studies were from European backgrounds. Further details on the GWAS datasets were presented in Table 1.

**Table 1:** Detailed information regarding studies and datasets used in the bidirectional MR analysis.

Phenotype	GWAS ID	Year	Sample size	nSNPs	Population
SOD	prot-a-2800	2018	3301	10,534,735	European
GST	prot-a-1283	2018	3301	10,534,735	European
GPX	prot-a-1265	2018	3301	10,534,735	European
CAT	prot-a-367	2018	3301	10,534,735	European
MPO	ebi-a-GCST90012031	2020	21,758	13,138,585	European
PON	ebi-a-GCST90010252	2020	1322	18,221,044	European
UA	ukb-d-30880_raw	2018	343,836	13,585,994	European
TBIL	ukb-d-30840_raw	2018	342,829	13,585,986	European
Retinol	ukb-b-17406	2018	62,991	9851,867	European
Albumin	met-d-Albumin	2020	115,060	12,321,875	European
Vitamin C	ukb-b-19390	2018	64,979	9851,867	European
Vitamin E	ukb-b-6888	2018	64,979	9851,867	European
Keratoconus	finn-b-H7_CORNEALDEFORM	2021	209,598	16,380,407	European

CAT = catalase, GPX = glutathione peroxidase, GST = glutathione transferase, GWAS = genome-wide association studies, MPO = myeloperoxidase, MR = Mendelian randomization, PON = paraoxonase, SNPs = single-nucleotide polymorphisms, SOD = super oxide dismutase, TBIL = total bilirubin, UA = uric acid.

### 2.3. IV selection

We conducted a sequence of screening procedures featuring rigorous quality control measures to pinpoint valid single-nucleotide polymorphisms (SNPs) for the IVs. Firstly, a correlative threshold was set at *P* < 5 × 10^−6^ as the screening condition to obtain enough SNPs strongly linked to exposures from the GWAS pooled database. Secondly, all selected SNPs were clumped and removed ineligible variants for the linkage disequilibrium using the PLINK algorithm (*r*^2^ < 0.001, clumping distance = 10,000 kilobases). Thirdly, the F-statistics were calculated for each SNP instrument with the formula *F* = Beta^2^/SE^2^.^[17]^ SNPs with *F*-statistics < 10 were used to filter all weak IVs and excluded from the analysis to minimize bias.^[18]^ This process guaranteed that the IVs employed in our MR analysis were robust and capable of supplying reliable causal inferences.

### 2.4. Statistical analysis

In order to explore the possible causal association between OSIBs and KC, inverse variance weighted (IVW) was adopted as the dominant analysis method, while the results of MR-Egger, simple mode, weighted median and weighted mode were selected as supplementary information. These methods were designed to evaluate causal relationships from different perspectives, ensuring multi-angle verification of the results. To delve deeply into the robustness of our results against potential pleiotropic effects, we conducted a sequence of sensitivity analyses. Cochran *Q* test was applied to evaluate the heterogeneity of the MR assumptions, with *P* > .05 suggesting the absence of heterogeneity. MR-Egger intercept test and MR-PRESSO (MR pleiotropy residual sum and outlier) were used to detect the presence of horizontal pleiotropy, with *P* > .05 indicating the absence of pleiotropy. Additionally, “leave-one-out” analyses were carried out to further explore genetic variants which might be impacted by a single strong SNP.

The statistical analyses mentioned above were conducted utilizing the specialized packages Two-Sample MR (version 0.6.17, China) and MR-PRESSO (version 1.0) in the R software (version 4.5.1, China). Given that the risk of KC was binary variables, we applied the odds ratios (ORs) and 95% confidence intervals (CIs) to illustrate the causal effects between OSIBs and KC. If the *P*-value was <.05, a potential causal association was deemed statistically significant.

## 3. Results

### 3.1. Genetic instrumental variables screening

Twelve OSIBs were assessed for the determination of their potential causal links with KC. A range of 7 to 372 SNPs was employed in the study. The *F*-statistics for all SNPs exceeded the threshold of 20, demonstrating strong instrument strength and ensuring the reliability of our MR estimates. Detailed summary statistics of these related SNPs were provided in Tables S1 and S2, Supplemental Digital Content, https://links.lww.com/MD/Q688.

### 3.2. Causal effect of genetically predicted OSIBs on KC

**Figure 2.**
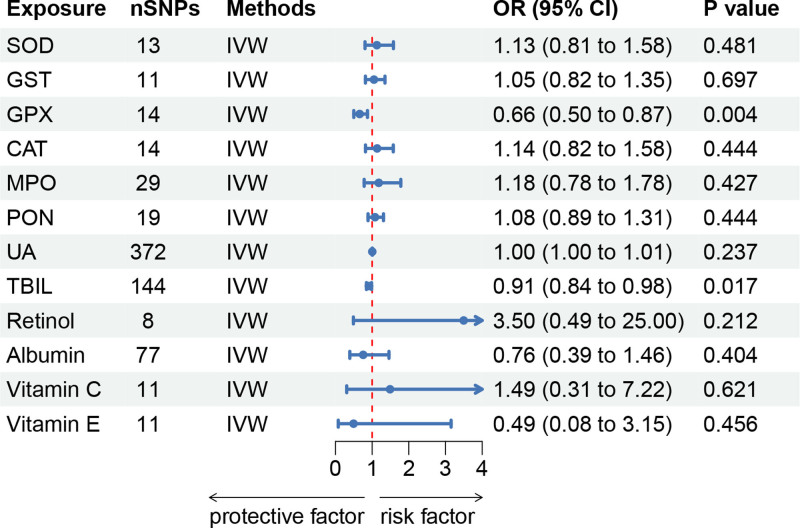
Forest plot for the causal effects of OSIBs on KC by the IVW method. CI = confidence interval, IVW = inverse variance weighted, OR = odds ratio, OSIBs = oxidative stress injury biomarkers, SNPs = single-nucleotide polymorphisms.

The IVW approach yielded findings concerning the causal relationship between OSIBs and KC. Figure 2 presents the forest plots of the associations between OSIBs and KC. The IVW method showed inverse associations between genetically predicted levels of GPX and diminished KC risk (OR: 0.660, 95% CI: 0.500–0.873, *P* = .004). However, the potential link between GPX and KC was no longer evident when using the other 4 methods. TBIL was also found to be associated with a decreased risk of KC through the IVW method (OR: 0.912, 95% CI: 0.845–0.984, *P* = .017), which was consistent with the result from the MR-Egger method (OR: 0.910, 95% CI:0.835–0.992, *P* = .034). There were no associations of SOD, GST, CAT, MPO, PON, UA, retinol, albumin, vitamin C, or vitamin E with KC. More information on the outcomes of the additional forward MR was presented in Table S3, Supplemental Digital Content, https://links.lww.com/MD/Q688.

**Figure 3.**
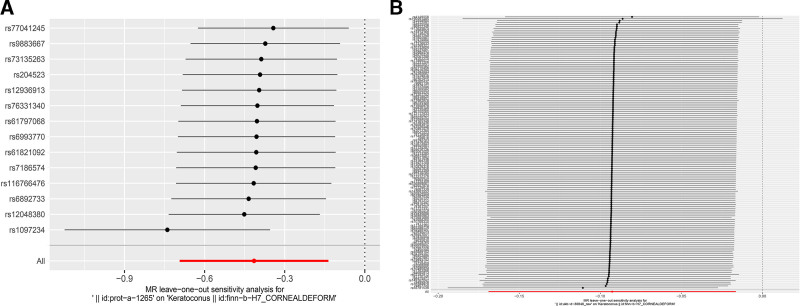
Leave-one-out plots of the causal effects of OSIBs on KC. (A) MR leave-one-out sensitivity analysis for GPX on KC. (B) MR leave-one-out sensitivity analysis for TBIL on KC. KC = keratoconus, OSIBs = oxidative stress injury biomarkers, TBIL = total bilirubin.

Sensitivity analyses were performed for OSIBs through Cochran *Q* test, MR-Egger intercept, and MR-PRESSO methodology (Table 2). Significant heterogeneity was detected between UA and KC by Cochran *Q* test (*P* = .033). Both MR-Egger intercept and MR-PRESSO analyses demonstrated the absence of horizontal pleiotropy in other OSIBs, apart from the linkage between UA and KC (MR-PRESSO *P* = .028). Leave-one-out analysis verified that the MR results were not prominently affected by individual SNPs, affirming the reliability and stability of the findings (Fig. 3).

**Table 2:** Pleiotropy and heterogeneity test of the forward MR analysis.

			Horizontal pleiotropy test			Heterogeneity test (IVW)				MR-PRESSO
				MR-Egger		IVW		MR-Egger		
Exposure	Outcome	nSNPs	Intercept	SE	*P*_intercept	*Q*	*Q*_pval	*Q*	*Q*_pval	Global test *P*
SOD	Keratoconus	13	5.48E‐02	0.077	.490	6.928	0.862	6.417	0.844	.860
GST		11	‐6.95E‐02	0.080	.405	9.786	0.459	9.022	0.435	.531
GPX		14	‐3.48E‐02	0.063	.591	13.458	0.413	13.125	0.360	.277
CAT		14	1.65E‐01	0.086	.081	13.235	0.430	9.603	0.651	.452
MPO		29	2.11E‐02	0.034	.536	30.261	0.351	29.827	0.322	.392
PON		19	5.60E‐02	0.070	.437	20.910	0.284	20.157	0.266	.253
UA		372	2.82E‐03	0.009	.761	422.452	0.033	422.346	0.031	.028
TBIL		144	7.79E‐04	0.010	.938	150.840	0.310	150.834	0.290	.334
Retinol		8	8.80E‐02	0.107	.444	4.926	0.669	4.254	0.642	.738
Albumin		77	‐4.22E‐03	0.021	.840	79.116	0.381	79.073	0.352	.406
Vitamin C		11	7.61E‐02	0.072	.315	10.636	0.387	9.448	0.397	.383
Vitamin E		11	1.00E‐01	0.083	.258	15.523	0.114	13.356	0.147	.140

CAT = catalase, GPX = glutathione peroxidase, GST = glutathione transferase, IVW = inverse variance weighted, MPO = Myeloperoxidase, MR = Mendelian randomization, MR-PRESSO = MR pleiotropy residual sum and outlier, PON = paraoxonase, SE = standard error, SNPs = single-nucleotide polymorphisms, SOD = super oxide dismutase, TBIL = total bilirubin, UA = uric acid.

### 3.3. Reverse MR analysis

**Figure 4.**
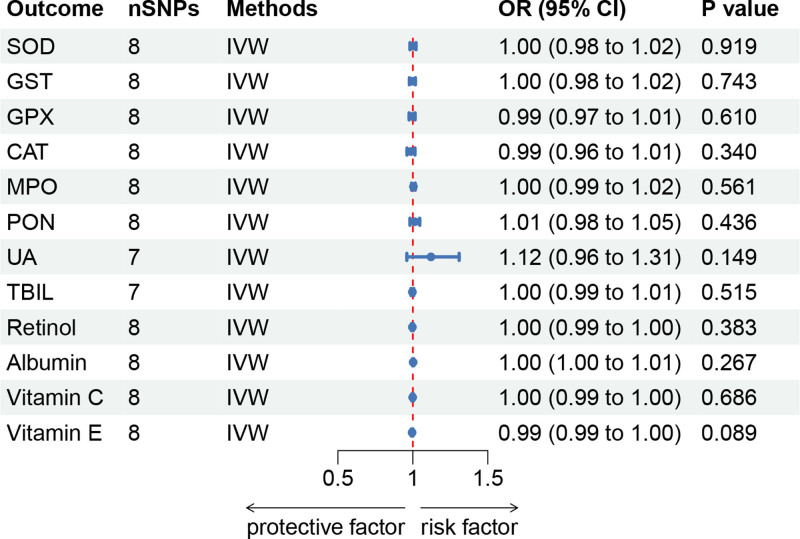
Forest plot for the causal effects of KC on OSIBs by the IVW method. CI = confidence interval, IVW = inverse variance weighted, KC = keratoconus, OR = odds ratio, OSIBs = oxidative stress injury biomarkers, SNPs = single-nucleotide polymorphisms.

Reverse MR analysis was conducted to explore the possible causal impacts of KC on OSIBs. The results of the MR analysis did not show any evidence of reverse causality (*P* > .05). The findings were provided in Figure 4 and Table S4, Supplemental Digital Content, https://links.lww.com/MD/Q688.

Sensitivity analyses of the reverse MR analysis revealed no evidence of pleiotropy and heterogeneity (*P* > .05), supporting the robustness and consistency of the causal estimates (Table S5, Supplemental Digital Content, https://links.lww.com/MD/Q688). Taken together, all of the findings indicated that genetically predicted KC had no causal effect on the 12 OSIBs considered in this study.

## 4. Discussion

In this study, we performed a bidirectional 2-sample MR analysis to evaluate the causal relationship between 12 OSIBs and KC. Our results indicated that OSIBs, particularly GPX and TBIL, exhibited significant associations with KC. Higher genetically predicted GPX and TBIL levels were linked to a reduced risk of KC, suggesting that both GPX and TBIL may act as protective factors against KC. These findings shed light on the underlying mechanisms of KC, offering valuable insights into its pathogenesis. Apart from researching the effect of OSIBs on KC, we also investigated reverse causality by analyzing the effect of KC on OSIBs. Our results did not show any evidence of reverse causality.

Increasing evidence have pointed out that OS is closely related to KC. Studies indicated that KC samples exhibit elevated OSIBs, such as CAT, extracellular superoxide dismutase, and malondialdehyde, alongside diminished antioxidant enzyme activity compared with healthy individuals. This imbalance compromised the ability of cornea to neutralize reactive oxygen species (ROS) and protected against oxidative damage.^[19]^ Moreover, multilevel analyses of tears, cornea, and cultured cells consistently revealed oxidative stress dysregulation and defective antioxidant mechanisms in KC.^[20]^ As far as we know, this is the first research to apply MR analysis to comprehensively estimate the causality association between OSIBs and KC. Despite the challenges posed by randomized controlled trials, this research provides genetic evidence supporting the prevention of KC by targeting OSIBs.

Although the antioxidant system of mitochondria can reduce excessive ROS, this capacity is relatively constrained. Therefore, antioxidants are indispensable for our body to combat oxidative damage.^[21]^ Enzymatic antioxidants, including SOD, GST, GPX, CAT, MPO, and PON, have the capacity to impede peroxides formation and eliminate free radicals. GPX is an enzymatic antioxidant that can decrease H2O2 and lipid hydroperoxides by utilizing reduced glutathione as a cofactor, which plays a crucial role in sustaining the balance of redox processes.^[22]^ Balmus IM et al discovered that the GPX enzyme activity in KC patients was notably increased compared with the control group, indicating a compensatory response to strong lipid peroxidation.^[23]^ Several researches investigated the relationship between genetic polymorphisms of GPX-1 and susceptibility to KC, which revealed a possible association between GPX-1 rs1050450 polymorphism and an increased risk of KC.^[9,24]^ These observational research results contradict the findings in our study. The observed inconsistencies may be attributed to disparities in study protocols and sample sizes. In addition, differences in the study population, including genetic background, environmental exposure, and dietary habits, may also affect the results. The intricate relationship between genetic factors and environmental impacts is highlighted in KC.^[25]^

Nonenzymatic antioxidants, including UA, TBIL, retinol, albumin, vitamin C, and vitamin E, constitute the secondary defense line of the antioxidant system and perform indispensable functions. They can rapidly scavenge free radicals and resist oxidants.^[26]^ TBIL is considered as a protective factor for KC in this study, which has been reported to possess robust antioxidant properties.^[27]^ The protective effect of TBIL against KC may be ascribed to a bunch of biological mechanisms. First of all, TBIL is a potent nonenzymatic antioxidant that can neutralize ROS and inhibit the activity of nicotinamide adenine dinucleotide phosphate oxidase, eventually reducing OS.^[28]^ Inflammation and OS imbalance may be key to the pathogenesis of KC.^[29]^ TBIL has the potential to inhibit inflammatory processes through its ability to scavenge ROS. Besides, TBIL has recently been regarded as an immunomodulatory molecule with powerful antioxidant and anti‐inflammatory properties.^[30]^ It can impede the activation of pro-inflammatory cytokines and modulate immune responses, thereby aiding in the suppression of KC development. On the other hand, TBIL is a product of heme oxygenase activity, which has been shown to play a protective role in various inflammatory disease models.^[31]^ The induction of heme oxygenase may signify a physiological reaction intended to alleviate the intensity of inflammation in KC.^[32]^ Although the relationship between TBIL and KC remains largely unexplored, further investigation is necessary to elucidate the precise mechanism through which TBIL influences KC and to pinpoint potential therapeutic targets for intervention.

The main strength of the present study lies in the utilization of MR analysis to explore the causal relationship between OS and the risk of KC, which effectively resolves reverse causality and minimizes the influence of confounding factors that frequently occur in observational researches. Including 12 OSIBs made it the most comprehensive MR study investigating the association between OS and KC. Furthermore, the incorporation of IVs originated solely from the European demographic diminished the possible impact of population stratification and improved the credibility of the MR analysis.

However, this study has several limitations: Firstly, to maintain adequate statistical efficiency of the MR analysis, the *P*-value threshold of GWAS was set at 5 × 10^−6^, consequently resulting in a relatively a small proportion of variance explained by the connections between IVs and specific exposures. Secondly, substantial imbalance between the control group (209,287) and the cases (311) presented several challenges and underlying biases for the research analysis. The presence of such striking differences could result in statistical imbalance, which could weaken the robustness of effect estimation and complicate the identification of genuine associations or causal relationships. Thirdly, we couldn’t evaluate the nonlinear causal link between OSIBs and KC due to the absence of individual data. Finally, as our MR analysis was conducted exclusively in individuals of European ancestry, the universality of findings from other bloodline groups might be constrained. Future studies should take into account different ethnic backgrounds and larger sample sizes to expand the applicability and reliability of the research results.

## 5. Conclusion

This study offers compelling evidence supporting the existence of a causal link between OSIBs and KC. Specifically, higher GPX and TBIL levels were associated with a reduced risk of KC. These findings emphasize the importance of OS in KC pathogenesis and provide potential biomarkers for early detection and targeted prevention. Further investigation is required to delve into the clinical significance of these biomarkers and comprehensively scrutinize the potential biological mechanisms.

## Acknowledgments

The authors wish to acknowledge the staff members who manage the IEU OpenGWAS database and the participants in data collection.

## Author contributions

**Conceptualization:** Jiao Wang, Jie Zhang, Qianqian Sun.

**Data curation:** Jiao Wang, Jie Zhang.

**Formal analysis:** Jiao Wang.

**Investigation:** Jiao Wang.

**Methodology:** Jiao Wang.

**Project administration:** Jiao Wang.

**Resources:** Jiao Wang.

**Software:** Jie Zhang.

**Supervision:** Jiao Wang, Qianqian Sun.

**Validation:** Jiao Wang, Qianqian Sun.

**Visualization:** Jiao Wang.

**Writing – original draft:** Jiao Wang, Jie Zhang.

**Writing – review & editing:** Qianqian Sun.

## Supplementary Material


