# Supplementary material for: The causal effect of oxidative stress on the risk of keratoconus: A bidirectional two-sample Mendelian randomization study
Source: Medicine (Baltimore). 2025 Dec 19;104(51):e46055. doi: 10.1097/MD.0000000000046055 (PMC12727323; doi:10.1097/MD.0000000000046055)
Supplement: Supplementary file 1 [file medi-104-e46055-s001.pdf]

**Supplementary Table S1. Characteristics of SNPs associated with 12 OSIB.**

| SNPs | EA | OA | Beta | EAF | Pval | SE | F |
|---|---|---|---|---|---|---|---|
| **1.SOD-Keratoconus** | | | | | | | |
| rs10745788 | T | A | -0.3101 | 0.9629 | 4.57088E-06 | 0.0677 | 20.9682886418266 |
| rs113089419 | C | A | 0.3207 | 0.04037 | 1.34896E-06 | 0.0664 | 23.3130208631305 |
| rs116530051 | A | C | 0.4002 | 0.02109 | 2.95121E-06 | 0.0856 | 21.8445664897493 |
| rs11861986 | T | G | -0.1359 | 0.76491 | 4.89779E-06 | 0.0297 | 20.9248718074969 |
| rs148622725 | T | C | 0.7258 | 0.00729 | 1.94984E-06 | 0.1526 | 22.6079743465009 |
| rs2016081 | C | T | -0.4437 | 0.01979 | 2.04174E-06 | 0.0934 | 22.5539127582615 |
| rs2695234 | G | A | -0.3427 | 0.90931 | 8.31764E-14 | 0.0459 | 55.7108300278461 |
| rs3790028 | C | G | -0.1721 | 0.12249 | 3.46737E-06 | 0.0371 | 21.505557854417 |
| rs4619877 | A | G | -0.1522 | 0.28589 | 7.94328E-08 | 0.0284 | 28.703140443456 |
| rs4803050 | A | T | 0.1501 | 0.23219 | 8.31764E-07 | 0.0305 | 24.2046327169839 |
| rs4856490 | C | T | -0.1558 | 0.18032 | 1.02329E-06 | 0.0319 | 23.8391261481792 |
| rs73164157 | C | A | 0.4627 | 0.0169 | 1.14815E-06 | 0.0951 | 23.6578218080251 |
| rs7523327 | A | G | -0.1164 | 0.4987 | 2.81838E-06 | 0.0249 | 21.839568711259 |
| **2.GST-Keratoconus** | | | | | | | |
| rs117829315 | G | T | -0.616 | 0.01305 | 1.31826E-06 | 0.1274 | 23.3646542943426 |
| rs12214820 | A | G | 0.1825 | 0.17638 | 9.77237E-08 | 0.0342 | 28.4583893411107 |
| rs2185401 | T | C | -0.35 | 0.03154 | 8.12831E-07 | 0.071 | 24.2860107253812 |
| rs2290758 | A | G | 0.4157 | 0.57002 | 7.24436E-69 | 0.0237 | 307.468159520473 |
| rs60042638 | A | G | 0.2116 | 0.10385 | 2.39883E-06 | 0.0449 | 22.196036787317 |
| rs73073444 | T | C | -0.5614 | 0.01095 | 2.51189E-06 | 0.1193 | 22.1309838101379 |

| | | | | | | | |
|---|---|---|---|---|---|---|---|
| rs75534686 | T | C | 0.4056 | 0.02338 | 3.01995E-06 | 0.0869 | 21.7717395826309 |
| rs7720426 | A | G | -0.15 | 0.18001 | 3.63078E-06 | 0.0324 | 21.4204844605846 |
| rs7819300 | T | C | -0.122 | 0.38504 | 2.18776E-06 | 0.0258 | 22.3468873965331 |
| rs78409028 | A | G | 0.7853 | 0.00605 | 1.14815E-06 | 0.1614 | 23.6592473853619 |
| rs79104405 | A | G | 0.4456 | 0.0236 | 4.67735E-08 | 0.0816 | 29.8020979685184 |
| **3.GPX-Keratoconus** | | | | | | | |
| rs1097234 | A | C | 0.5633 | 0.17474 | 1.62181E-73 | 0.0311 | 327.865345737944 |
| rs116766476 | T | G | 0.5526 | 0.01172 | 4.7863E-06 | 0.1207 | 20.9480697931923 |
| rs12048380 | C | T | -0.173 | 0.85412 | 3.71535E-06 | 0.0374 | 21.3838447675502 |
| rs12936913 | T | C | -0.127 | 0.28207 | 3.0903E-06 | 0.0272 | 21.7874510836865 |
| rs204523 | T | A | -0.15 | 0.81078 | 1.94984E-06 | 0.0315 | 22.6619982538103 |
| rs61797068 | C | G | 0.1589 | 0.15569 | 3.63078E-06 | 0.0343 | 21.4484713718067 |
| rs61821092 | G | T | -0.2171 | 0.14059 | 9.54993E-08 | 0.0407 | 28.4359419957247 |
| rs6892733 | A | G | 0.1133 | 0.54018 | 3.98107E-06 | 0.0246 | 21.1995380122745 |
| rs6993770 | T | A | -0.134 | 0.28225 | 1.04713E-06 | 0.0274 | 23.9026065220082 |
| rs7186574 | T | C | 0.1706 | 0.15073 | 1.51356E-06 | 0.0355 | 23.0801240527463 |
| rs73135263 | G | C | -0.4228 | 0.02034 | 4.16869E-06 | 0.0918 | 21.199293425033 |
| rs76331340 | A | G | -0.3168 | 0.04933 | 1.38038E-07 | 0.0601 | 27.768869085749 |
| rs77041245 | A | G | -0.579 | 0.01077 | 2.34423E-06 | 0.1227 | 22.2538313899172 |
| rs9883667 | A | G | -0.284 | 0.04044 | 4.36516E-06 | 0.0618 | 21.1055425645223 |
| **4.CAT-Keratoconus** | | | | | | | |
| rs10918963 | C | T | -0.1459 | 0.20245 | 2.45471E-06 | 0.031 | 22.137266192788 |
| rs116670019 | T | C | -0.4967 | 0.01787 | 3.54813E-06 | 0.1071 | 21.4954315969857 |

| | | | | | | | |
|---|---|---|---|---|---|---|---|
| rs116970161 | T | G | 0.4723 | 0.01798 | 1.12202E-06 | 0.097 | 23.6934996979469 |
| rs13381678 | T | C | 0.1712 | 0.13178 | 2.88403E-06 | 0.0366 | 21.8666443341223 |
| rs1685815 | A | G | -0.1454 | 0.74594 | 9.12011E-07 | 0.0296 | 24.1147176968309 |
| rs17089624 | C | T | -0.2808 | 0.05216 | 5.01187E-07 | 0.0559 | 25.2178108179356 |
| rs2745924 | T | G | 0.1894 | 0.54506 | 2.69153E-14 | 0.0249 | 57.8226573150277 |
| rs28549749 | C | T | 0.1215 | 0.36781 | 3.31131E-06 | 0.0261 | 21.6575004655743 |
| rs4806509 | T | G | 0.1429 | 0.6723 | 6.76083E-07 | 0.0288 | 24.6045979734618 |
| rs4970968 | T | G | 0.1208 | 0.64165 | 2.88403E-06 | 0.0258 | 21.9094385177469 |
| rs56106813 | T | C | -0.257 | 0.0674 | 5.01187E-07 | 0.0511 | 25.2790784462249 |
| rs700715 | C | T | -0.1358 | 0.67052 | 2.23872E-07 | 0.0262 | 26.8493482815005 |
| rs73087640 | T | C | 0.1948 | 0.09741 | 2.5704E-06 | 0.0414 | 22.1265658247459 |
| rs79696893 | G | A | 0.3234 | 0.03394 | 4.7863E-06 | 0.0707 | 20.9111537288624 |
| **5.MPO-Keratoconus** | | | | | | | |
| rs10103048 | C | A | -0.0742 | 0.5849 | 1.14209E-14 | 0.0096 | 59.74001736 |
| rs10282219 | G | A | -0.0492 | 0.2887 | 2.94503E-06 | 0.0105 | 21.95591837 |
| rs10418923 | G | A | 0.0651 | 0.7081 | 7.49704E-10 | 0.0106 | 37.71813813 |
| rs10753459 | A | G | 0.1092 | 0.1753 | 1.43285E-19 | 0.0121 | 81.4468957 |
| rs11199938 | C | T | -0.0575 | 0.3474 | 2.19801E-07 | 0.0111 | 26.8342667 |
| rs1150754 | T | C | -0.0748 | 0.152 | 5.873E-09 | 0.0128 | 34.14941406 |
| rs117148585 | A | T | 0.148 | 0.0345 | 6.78594E-07 | 0.0298 | 24.66555561 |
| rs12589195 | A | C | -0.0568 | 0.2446 | 1.64801E-07 | 0.0109 | 27.15461661 |
| rs13032049 | G | A | -0.0496 | 0.2777 | 2.93197E-06 | 0.0106 | 21.89533642 |
| rs13107325 | T | C | 0.1078 | 0.0657 | 7.09905E-07 | 0.0217 | 24.67845994 |

| | | | | | | | |
|---|---|---|---|---|---|---|---|
| rs138285555 | G | C | 0.1646 | 0.0299 | 2.03002E-06 | 0.0347 | 22.50094262 |
| rs145096717 | A | G | 0.2792 | 0.0159 | 2.51698E-06 | 0.0593 | 22.16774113 |
| rs150918492 | A | G | -0.0722 | 0.2625 | 5.81005E-09 | 0.0124 | 33.90244537 |
| rs188737126 | G | A | 0.2022 | 0.0311 | 7.49808E-07 | 0.0409 | 24.44081516 |
| rs2167252 | G | C | -0.0533 | 0.7827 | 2.357E-06 | 0.0113 | 22.24833581 |
| rs25913 | C | T | 0.0494 | 0.3374 | 3.22797E-06 | 0.0106 | 21.71911712 |
| rs34097845 | T | C | -0.3492 | 0.0566 | 2.69712E-73 | 0.0193 | 327.3662112 |
| rs34723959 | A | G | 0.2092 | 0.0221 | 7.03396E-07 | 0.0422 | 24.57527908 |
| rs417463 | A | G | -0.0494 | 0.4697 | 3.22901E-07 | 0.0097 | 25.93644383 |
| rs4942786 | G | A | 0.0482 | 0.5164 | 4.20204E-06 | 0.0105 | 21.07247166 |
| rs564124209 | A | C | -0.3344 | 0.017 | 1.011E-07 | 0.0628 | 28.35392917 |
| rs6034875 | A | G | -0.0752 | 0.5824 | 3.99761E-15 | 0.0096 | 61.36111111 |
| rs62442278 | T | A | 0.0733 | 0.1874 | 1.80801E-07 | 0.0141 | 27.02525024 |
| rs66493857 | G | A | -0.0537 | 0.6724 | 4.60797E-06 | 0.0117 | 21.06574622 |
| rs74343467 | T | C | -0.0535 | 0.5443 | 3.97E-07 | 0.0106 | 25.4739231 |
| rs7502971 | A | C | -0.0559 | 0.3703 | 9.21892E-09 | 0.0097 | 33.21086194 |
| rs757081 | G | C | 0.0982 | 0.3355 | 4.26481E-23 | 0.0099 | 98.39036833 |
| rs78978525 | G | A | -0.1479 | 0.0418 | 4.20998E-07 | 0.0292 | 25.65491884 |
| rs8178414 | T | C | -0.4359 | 0.0218 | 4.85848E-29 | 0.039 | 124.9236095 |
| **6.PON-Keratoconus** | | | | | | | |
| rs112922902 | A | G | -0.264992 | 0.18 | 6.53898E-07 | 0.0523689 | 25.60463696 |
| rs114910051 | C | G | 0.539785 | 0.033 | 3.23631E-06 | 0.114381 | 22.27069886 |
| rs116612893 | A | G | -0.534347 | 0.034 | 4.26265E-06 | 0.114458 | 21.79487814 |

| | | | | | | | |
|---|---|---|---|---|---|---|---|
| rs1359646 | C | T | -0.206659 | 0.714 | 2.29256E-06 | 0.0431991 | 22.88543908 |
| rs1371674 | T | G | 0.209676 | 0.689 | 2.40963E-06 | 0.043921 | 22.79045557 |
| rs140691177 | T | G | -1.02945 | 0.012 | 3.16665E-08 | 0.18236 | 31.86774614 |
| rs149867961 | C | T | -1.18444 | 0.031 | 7.57879E-23 | 0.112846 | 110.1675756 |
| rs149956071 | A | G | -0.999342 | 0.012 | 1.04609E-07 | 0.184949 | 29.19606389 |
| rs16832400 | G | A | 0.669172 | 0.025 | 2.08718E-07 | 0.126849 | 27.82924508 |
| rs183048297 | T | C | -1.05962 | 0.028 | 3.61243E-17 | 0.120672 | 77.10583991 |
| rs2927710 | C | T | 0.29215 | 0.156 | 1.16453E-07 | 0.0541993 | 29.05523726 |
| rs421510 | C | T | 0.201166 | 0.418 | 1.03124E-06 | 0.0406314 | 24.51238604 |
| rs62240714 | T | C | 0.361412 | 0.077 | 4.67046E-06 | 0.0774057 | 21.80013846 |
| rs6503361 | C | A | -0.761343 | 0.985 | 3.597E-06 | 0.161871 | 22.12190988 |
| rs73227481 | G | C | -0.7533 | 0.039 | 1.45781E-11 | 0.108443 | 48.25395199 |
| rs75951132 | T | C | -0.358556 | 0.082 | 1.62795E-06 | 0.0737116 | 23.66150214 |
| rs76122431 | A | G | -0.422959 | 0.055 | 2.03531E-06 | 0.0879098 | 23.14845218 |
| rs77885169 | C | T | -0.763459 | 0.034 | 2.1419E-11 | 0.111066 | 47.25080108 |
| rs7866750 | A | G | 0.222023 | 0.237 | 3.19823E-06 | 0.0470145 | 22.30140834 |
| **7.UA-Keratoconus** | | | | | | | |
| rs10064782 | G | A | 1.225 | 0.25305 | 9.96552E-11 | 0.18941 | 41.8276863807093 |
| rs10131425 | C | T | -1.045 | 0.2061 | 3.27537E-07 | 0.20462 | 26.0815798202266 |
| rs10160397 | C | T | 0.88891 | 0.39265 | 1.21711E-07 | 0.168 | 27.9959039097782 |
| rs10164318 | G | T | -1.5901 | 0.67313 | 9.96552E-20 | 0.17493 | 82.6261955855427 |
| rs10174267 | A | T | 0.83536 | 0.63263 | 9.17656E-07 | 0.17018 | 24.0950475068541 |

| | | | | | | | |
|---|---|---|---|---|---|---|---|
| rs10193587 | C | T | -1.0803 | 0.23066 | 3.86741E-08 | 0.19653 | 30.2154118110505 |
| rs10196697 | A | G | -0.93225 | 0.39092 | 3.02907E-08 | 0.16828 | 30.6900256353087 |
| rs10210970 | T | C | 1.4434 | 0.12894 | 3.61676E-09 | 0.24461 | 34.8194943897363 |
| rs10224210 | C | T | 2.1792 | 0.28247 | 6.17732E-33 | 0.18228 | 142.926781603457 |
| rs10279504 | A | C | -1.0933 | 0.24141 | 1.21809E-08 | 0.1919 | 32.4583336769785 |
| rs10405423 | A | C | 1.83 | 0.65781 | 1.09901E-25 | 0.17465 | 109.789921190034 |
| rs1047891 | A | C | -2.1358 | 0.31573 | 9.61612E-34 | 0.17638 | 146.629030019707 |
| rs10507059 | T | C | 1.3734 | 0.23171 | 1.50176E-12 | 0.19413 | 50.0502499700368 |
| rs1051453 | T | C | -0.78238 | 0.41418 | 2.61631E-06 | 0.1665 | 22.0802750092167 |
| rs10754894 | A | G | -1.7524 | 0.31406 | 3.04579E-23 | 0.17644 | 98.6438160417585 |
| rs10777836 | G | A | 0.8875 | 0.28009 | 1.17411E-06 | 0.18261 | 23.6202823277337 |
| rs10782230 | A | G | 1.2167 | 0.48093 | 1.24796E-13 | 0.16415 | 54.9392486611346 |
| rs10784330 | G | A | 0.92795 | 0.32628 | 1.22699E-07 | 0.17543 | 27.9794323527115 |
| rs10797999 | T | C | -0.99748 | 0.41098 | 2.36102E-09 | 0.16706 | 35.6501131752428 |
| rs10817881 | G | A | 0.81418 | 0.6672 | 2.94327E-06 | 0.17416 | 21.854531535329 |
| rs10846156 | G | T | -1.2182 | 0.20201 | 2.67621E-09 | 0.20472 | 35.4090372234887 |
| rs1087925 | C | G | -1.5911 | 0.075193 | 3.2276E-07 | 0.31139 | 26.1085710043497 |
| rs10886117 | A | G | 1.5703 | 0.16706 | 1.01228E-12 | 0.22027 | 50.8220327366833 |
| rs10899125 | G | T | 1.8737 | 0.071184 | 1.8189E-08 | 0.3329 | 31.6788930047391 |
| rs10901057 | G | C | 1.725 | 0.919826 | 2.38199E-08 | 0.30904 | 31.1563290842983 |
| rs10907227 | C | G | 0.82749 | 0.40786 | 7.04206E-07 | 0.16682 | 24.6051914395471 |
| rs10914262 | A | C | 0.9363 | 0.25241 | 7.31611E-07 | 0.18904 | 24.5312740015389 |

| | | | | | | | |
|---|---|---|---|---|---|---|---|
| rs10922199 | A | G | 0.8621 | 0.32874 | 1.13551E-06 | 0.17714 | 23.685332363271 |
| rs10926512 | G | A | 0.76848 | 0.40672 | 4.3383E-06 | 0.16726 | 21.1095233694998 |
| rs10933713 | G | C | 0.98758 | 0.29781 | 3.913E-08 | 0.17973 | 30.1926267141286 |
| rs10935686 | C | T | -1.0102 | 0.56061 | 9.37648E-10 | 0.16508 | 37.4475390014009 |
| rs10958567 | G | C | -1.2838 | 0.24463 | 1.61287E-11 | 0.19054 | 45.3962411903015 |
| rs10972546 | T | G | -1.3445 | 0.12233 | 9.03025E-08 | 0.25153 | 28.5719252540649 |
| rs11030084 | T | C | -0.98212 | 0.18611 | 3.12759E-06 | 0.21065 | 21.7372028001669 |
| rs11056337 | G | A | -0.83252 | 0.42137 | 5.56365E-07 | 0.16631 | 25.058213075323 |
| rs11072567 | G | A | 2.4491 | 0.51215 | 2.05353E-50 | 0.16399 | 223.036416489002 |
| rs11128603 | G | A | -1.7258 | 0.12134 | 5.94292E-12 | 0.25079 | 47.354142011687 |
| rs111346856 | A | G | 1.0961 | 0.27703 | 2.2519E-09 | 0.18333 | 35.7462717343252 |
| rs11136326 | G | A | 0.86299 | 0.67299 | 1.23689E-06 | 0.17795 | 23.5186839370069 |
| rs11158605 | A | T | -0.95413 | 0.6633 | 3.9355E-08 | 0.17367 | 30.1830392010196 |
| rs11163481 | T | G | 1.0249 | 0.67037 | 4.61158E-09 | 0.17487 | 34.350245081668 |
| rs11202328 | T | C | -1.9962 | 0.13132 | 2.19381E-16 | 0.2431 | 67.4272827856141 |
| rs11218783 | A | G | 0.91109 | 0.43061 | 4.13666E-08 | 0.16611 | 30.083507983763 |
| rs11238529 | G | C | 2.208 | 0.032026 | 2.1301E-06 | 0.46575 | 22.4744920421834 |
| rs11243632 | T | A | -1.2455 | 0.14325 | 1.30659E-07 | 0.23598 | 27.8570128149827 |
| rs112558796 | T | C | 3.0434 | 0.03127 | 1.25681E-10 | 0.47313 | 41.3765783289693 |
| rs11261022 | A | C | -0.81401 | 0.35557 | 2.0006E-06 | 0.17125 | 22.5941388754832 |
| rs113209314 | C | T | -1.0984 | 0.35782 | 1.38621E-10 | 0.17115 | 41.1874320385769 |
| rs114165349 | C | G | 6.4691 | 0.023533 | 1.51635E-32 | 0.54452 | 141.14241082218 |
| rs11564722 | T | C | -2.0452 | 0.23803 | 1.00415E-25 | 0.19503 | 109.967968056497 |
| rs116402366 | A | G | 4.4842 | 0.025027 | 7.19449E-17 | 0.5374 | 69.6261046168384 |

| | | | | | | | |
|---|---|---|---|---|---|---|---|
| rs11663020 | A | G | -0.7857 | 0.56359 | 1.98381E-06 | 0.16523 | 22.6116978939673 |
| rs11666281 | T | C | 1.3829 | 0.25376 | 2.09701E-13 | 0.18834 | 53.913049158753 |
| rs11693363 | C | A | -2.4112 | 0.13087 | 3.5156E-23 | 0.24312 | 98.360917155791 |
| rs11709427 | T | C | 2.6366 | 0.42412 | 1.36867E-56 | 0.1663 | 251.363069375491 |
| rs1171614 | C | T | 4.495 | 0.76883 | 5.99791E-118 | 0.19461 | 533.490340824527 |
| rs11736243 | T | C | -18.765 | 0.010844 | 5.90201E-124 | 0.79214 | 561.165190083369 |
| rs11748431 | A | G | -1.0374 | 0.2619 | 3.13473E-08 | 0.18747 | 30.6214958738575 |
| rs11757670 | T | C | -1.0444 | 0.15101 | 4.98907E-06 | 0.22877 | 20.841695944133 |
| rs118167925 | G | C | -1.8359 | 0.044965 | 3.60836E-06 | 0.39628 | 21.4630387102647 |
| rs11835818 | C | T | -1.4378 | 0.47895 | 2.21564E-18 | 0.16439 | 76.496865742983 |
| rs11854957 | T | C | -1.1463 | 0.21878 | 7.20892E-09 | 0.19811 | 33.4796772511646 |
| rs11871152 | G | A | 0.8807 | 0.48135 | 8.39383E-08 | 0.16435 | 28.7153457405196 |
| rs11940694 | G | A | 0.90012 | 0.60643 | 1.08131E-07 | 0.16943 | 28.2239422715696 |
| rs11948950 | A | G | 1.316 | 0.10516 | 1.75991E-06 | 0.27536 | 22.8406020089571 |
| rs12133907 | A | C | 1.5561 | 0.6023 | 1.40249E-20 | 0.16731 | 86.5025043176857 |
| rs12144369 | T | C | -0.97914 | 0.35829 | 2.03972E-08 | 0.17457 | 31.4592116755952 |
| rs12277177 | G | A | 1.285 | 0.17535 | 2.47201E-09 | 0.21547 | 35.5655972230707 |
| rs1229984 | C | T | -5.0278 | 0.977726 | 1.47096E-19 | 0.55571 | 81.8572286475579 |
| rs12316443 | A | C | -1.2554 | 0.30511 | 2.55976E-12 | 0.17934 | 49.0012762640025 |
| rs12363578 | T | C | -4.4467 | 0.4301 | 6.53131E-156 | 0.16707 | 708.396145506214 |
| rs12364728 | A | G | 2.4789 | 0.028769 | 3.37598E-06 | 0.5335 | 21.5897016713692 |
| rs12371604 | C | T | -1.0748 | 0.21843 | 5.98288E-08 | 0.19833 | 29.3681077959543 |

| | | | | | | | |
|---|---|---|---|---|---|---|---|
| rs12443147 | T | C | -1.1862 | 0.2135 | 3.73267E-09 | 0.20119 | 34.7616619593456 |
| rs12454490 | A | C | -1.4898 | 0.907082 | 1.68729E-07 | 0.28481 | 27.3616917600788 |
| rs12510175 | G | C | -0.93618 | 0.34269 | 6.61912E-08 | 0.17332 | 29.175535153261 |
| rs12542030 | G | C | -0.88999 | 0.62388 | 1.5372E-07 | 0.16958 | 27.543458129039 |
| rs12554192 | G | A | 1.154 | 0.26969 | 4.16534E-10 | 0.18471 | 39.0326713443311 |
| rs12576996 | G | T | 2.9004 | 0.24609 | 7.57356E-52 | 0.19141 | 229.606416328221 |
| rs12580556 | A | G | 1.2212 | 0.11087 | 2.98731E-06 | 0.2614 | 21.8252837458768 |
| rs12593988 | G | A | 1.0039 | 0.80828 | 1.74181E-06 | 0.20995 | 22.863705861186 |
| rs1260326 | C | T | -3.9953 | 0.60689 | 2.03236E-125 | 0.16765 | 567.922596648346 |
| rs12640131 | T | C | -1.0688 | 0.2673 | 8.33624E-09 | 0.18551 | 33.1937037190532 |
| rs12692751 | A | G | 0.90685 | 0.38288 | 8.04267E-08 | 0.16899 | 28.7969423249768 |
| rs12708477 | C | A | -1.1385 | 0.77225 | 5.99266E-09 | 0.19572 | 33.837099585698 |
| rs12716599 | C | G | 0.81744 | 0.32064 | 3.22931E-06 | 0.17558 | 21.675002444046 |
| rs12806743 | T | G | -1.3021 | 0.23683 | 2.22946E-11 | 0.19462 | 44.762178544347 |
| rs1285875 | C | G | -3.165 | 0.74254 | 1.14842E-63 | 0.18785 | 283.871964809788 |
| rs12888957 | G | C | 0.82735 | 0.30284 | 3.55279E-06 | 0.17846 | 21.4928614450282 |
| rs12908813 | T | C | 0.77916 | 0.5167 | 2.28929E-06 | 0.16486 | 22.3367601410358 |
| rs12979148 | C | T | 1.12 | 0.1305 | 4.30249E-06 | 0.24368 | 21.1248555117861 |
| rs13006913 | A | G | 1.0619 | 0.70554 | 3.49269E-09 | 0.17978 | 34.8884731279283 |
| rs13035759 | A | G | -0.85455 | 0.62088 | 4.52866E-07 | 0.16937 | 25.4565447398936 |
| rs13106497 | T | C | -1.2322 | 0.5997 | 1.90634E-13 | 0.16752 | 54.1036486443735 |
| rs13107325 | T | C | -2.5204 | 0.074844 | 6.66193E-16 | 0.31205 | 65.2361078245982 |

| | | | | | | | |
|---|---|---|---|---|---|---|---|
| rs13141123 | C | T | 6.8005 | 0.6381 | 1E-200 | 0.17132 | 1575.66189458249 |
| rs13153909 | A | T | 1.1654 | 0.67279 | 3.95276E-11 | 0.17641 | 43.6416439101785 |
| rs1317983 | C | T | 2.528 | 0.69388 | 5.93608E-46 | 0.17762 | 202.566620006673 |
| rs13222509 | T | C | -0.82392 | 0.529 | 5.2723E-07 | 0.16425 | 25.1626749604088 |
| rs13240994 | C | T | -2.8724 | 0.19956 | 1.41514E-44 | 0.20504 | 196.250207034965 |
| rs13316 | A | C | -0.89955 | 0.42974 | 9.05649E-08 | 0.1683 | 28.5680013465839 |
| rs13330604 | T | A | 2.2979 | 0.14715 | 2.70209E-23 | 0.23109 | 98.8774867201675 |
| rs13411042 | A | C | 1.3257 | 0.48462 | 6.97268E-16 | 0.16425 | 65.1444212206315 |
| rs139212650 | C | T | 3.3742 | 0.014135 | 2.86589E-06 | 0.72095 | 21.9042719956787 |
| rs139338026 | G | T | 1.6623 | 0.11339 | 1.34719E-10 | 0.25885 | 41.240122338425 |
| rs139721542 | A | G | 2.6215 | 0.022116 | 4.94493E-06 | 0.57399 | 20.8587628004486 |
| rs140181495 | C | T | -3.1294 | 0.018123 | 7.21905E-07 | 0.63151 | 24.5560871609728 |
| rs140254647 | C | T | -1.2554 | 0.21545 | 3.53427E-10 | 0.20011 | 39.3571949966846 |
| rs140275488 | C | A | 6.8544 | 0.012575 | 4.12762E-19 | 0.76722 | 79.8172080378534 |
| rs140696867 | T | C | -3.1154 | 0.016765 | 3.58319E-06 | 0.67225 | 21.4764988542571 |
| rs142768484 | T | C | -2.8372 | 0.020632 | 3.0715E-06 | 0.60806 | 21.7713071982228 |
| rs144006837 | A | G | 1.5059 | 0.085704 | 4.15891E-07 | 0.29752 | 25.6187189741219 |
| rs145148878 | T | C | 3.0873 | 0.01979 | 2.35229E-07 | 0.59725 | 26.7203918569247 |
| rs148136687 | G | A | 4.2571 | 0.0086225 | 3.33818E-06 | 0.91573 | 21.6117678240572 |
| rs148179165 | A | C | 1.3079 | 0.30611 | 2.90469E-13 | 0.17918 | 53.2804015023151 |
| rs148668346 | T | G | 3.8879 | 0.010923 | 3.02148E-06 | 0.83263 | 21.803365569195 |
| rs148951726 | T | G | -20.646 | 0.030565 | 1E-200 | 0.47544 | 1885.72294431493 |
| rs150147865 | T | A | -9.0477 | 0.0088936 | 3.1703E-22 | 0.93318 | 94.0032812130547 |

| | | | | | | | |
|---|---|---|---|---|---|---|---|
| rs1511299 | C | T | -1.7772 | 0.26077 | 1.92619E-21 | 0.18688 | 90.4366428110824 |
| rs151172114 | C | G | 4.223 | 0.024819 | 1.33999E-14 | 0.54828 | 59.3246013418602 |
| rs1520455 | T | C | -0.81691 | 0.61073 | 2.02488E-06 | 0.17194 | 22.5731440908961 |
| rs1570669 | G | A | 1.1091 | 0.34372 | 1.54721E-10 | 0.17327 | 40.9724688394644 |
| rs162000 | A | C | 0.79901 | 0.59323 | 2.65021E-06 | 0.17014 | 22.054085117347 |
| rs1636893 | G | C | -0.79214 | 0.37539 | 2.97557E-06 | 0.16953 | 21.8327397455334 |
| rs1680887 | T | C | -1.0351 | 0.77935 | 1.81589E-07 | 0.19839 | 27.2221571099579 |
| rs17024124 | T | A | 3.0694 | 0.021034 | 8.10214E-08 | 0.57212 | 28.7826112157779 |
| rs17024258 | T | C | 2.8314 | 0.02586 | 4.67401E-08 | 0.51825 | 29.8484172966617 |
| rs17050272 | A | G | 2.0284 | 0.40966 | 4.91813E-34 | 0.16676 | 147.952021932074 |
| rs17113991 | A | G | 1.9107 | 0.040995 | 3.9897E-06 | 0.41429 | 21.270298265953 |
| rs17183750 | A | G | 1.2228 | 0.17372 | 1.85609E-08 | 0.21738 | 31.6423816995238 |
| rs17396317 | A | G | -1.1831 | 0.13801 | 6.51914E-07 | 0.2378 | 24.7523639420162 |
| rs17553790 | G | A | 1.014 | 0.42485 | 1.70388E-09 | 0.16833 | 36.2869391935777 |
| rs17592117 | C | T | 3.4019 | 0.12515 | 3.12824E-42 | 0.24977 | 185.506876334918 |
| rs17624477 | C | T | 2.156 | 0.063891 | 1.2636E-10 | 0.33521 | 41.3676694719671 |
| rs17632159 | C | G | -2.6749 | 0.30419 | 1.15213E-50 | 0.17865 | 224.184991340824 |
| rs17817497 | C | T | 1.4525 | 0.39284 | 5.47646E-18 | 0.16805 | 74.7055250333182 |
| rs1800961 | T | C | -4.0106 | 0.031412 | 1.43516E-17 | 0.47002 | 72.8087368270118 |
| rs1806656 | G | C | 0.93148 | 0.32036 | 1.53349E-07 | 0.17747 | 27.5482898784178 |
| rs181673 | C | A | 1.536 | 0.53613 | 8.54083E-21 | 0.16421 | 87.4944897998155 |
| rs187355703 | G | C | 4.5313 | 0.025703 | 3.62076E-18 | 0.5214 | 75.526794422412 |

| | | | | | | | |
|---|---|---|---|---|---|---|---|
| rs1929926 | T | C | -0.94132 | 0.31923 | 9.34953E-08 | 0.17631 | 28.504810307414 |
| rs193220 | T | C | -1.2538 | 0.31734 | 1.21088E-12 | 0.17648 | 50.4734527492831 |
| rs195485 | T | C | -1.0613 | 0.38469 | 3.42673E-10 | 0.16904 | 39.4180226025302 |
| rs1955949 | C | T | -1.151 | 0.71306 | 2.1739E-10 | 0.1813 | 40.3044048786226 |
| rs1965132 | A | C | 1.2759 | 0.49442 | 1.25922E-14 | 0.16548 | 59.4483389181939 |
| rs1969977 | G | A | -1.9288 | 0.23029 | 5.00726E-22 | 0.1999 | 93.0992710014304 |
| rs1991371 | A | G | 2.0377 | 0.11941 | 7.80728E-16 | 0.25289 | 64.9254026774315 |
| rs2088054 | T | C | 1.1708 | 0.16327 | 1.3342E-07 | 0.22199 | 27.8160897501671 |
| rs2195525 | T | C | -1.176 | 0.52068 | 1.28027E-12 | 0.16572 | 50.3572804611563 |
| rs219787 | T | C | -1.7234 | 0.2628 | 2.30409E-20 | 0.18636 | 85.5193216797598 |
| rs2229357 | A | G | -3.8521 | 0.24372 | 1.17112E-90 | 0.19073 | 407.90080454209 |
| rs2240390 | C | T | -1.1469 | 0.3936 | 1.06365E-11 | 0.16871 | 46.2132943303339 |
| rs2258043 | C | T | -0.9791 | 0.49006 | 2.46383E-09 | 0.16417 | 35.5683712657673 |
| rs2345962 | G | A | 1.2004 | 0.49877 | 2.93292E-13 | 0.16449 | 53.2562332576577 |
| rs2357667 | T | C | 1.1027 | 0.14862 | 2.49488E-06 | 0.23418 | 22.1723956492798 |
| rs2437817 | A | C | -1.2079 | 0.33016 | 6.08415E-12 | 0.17563 | 47.3000972542824 |
| rs2439823 | G | A | 0.92963 | 0.54673 | 1.83451E-08 | 0.16521 | 31.662490472356 |
| rs2480714 | T | G | 1.1501 | 0.69836 | 2.14922E-10 | 0.1811 | 40.3303304165566 |
| rs2511656 | C | T | -1.68 | 0.85436 | 5.20475E-13 | 0.23268 | 52.1312246099087 |
| rs255751 | T | C | 1.5058 | 0.69907 | 4.59515E-17 | 0.17932 | 70.5138778592999 |
| rs2582309 | T | C | -0.82611 | 0.6703 | 2.19649E-06 | 0.17449 | 22.4146595627418 |
| rs2606228 | C | A | -0.86349 | 0.64293 | 6.83455E-07 | 0.17388 | 24.6611222223997 |

| | | | | | | | |
|---|---|---|---|---|---|---|---|
| rs2608930 | C | T | 1.0552 | 0.1806 | 7.86539E-07 | 0.21366 | 24.3904936780936 |
| rs2611774 | A | G | 1.0799 | 0.33559 | 5.12106E-10 | 0.17374 | 38.6335771256105 |
| rs2636590 | A | G | 0.90619 | 0.61313 | 7.62869E-08 | 0.16857 | 28.898508645739 |
| rs2648539 | C | T | -0.85812 | 0.62656 | 4.73108E-07 | 0.17036 | 25.3722077739712 |
| rs2676434 | G | A | 0.9607 | 0.68833 | 9.17445E-08 | 0.17982 | 28.542832476573 |
| rs2688 | T | G | -0.93214 | 0.60656 | 3.02489E-08 | 0.16825 | 30.6937264086387 |
| rs2695580 | A | G | 1.0008 | 0.41605 | 3.47568E-09 | 0.16941 | 34.8990803150267 |
| rs2701316 | A | G | 1.153 | 0.86678 | 1.8731E-06 | 0.24189 | 22.7206242453829 |
| rs2713210 | G | A | -1.2416 | 0.3602 | 4.06912E-13 | 0.17118 | 52.6083689819045 |
| rs2725202 | A | G | 10.987 | 0.10134 | 1E-200 | 0.27206 | 1630.89625478079 |
| rs2788144 | G | A | 3.1404 | 0.03673 | 7.04044E-13 | 0.43745 | 51.5359836224449 |
| rs2817188 | G | A | 5.3836 | 0.56825 | 1E-200 | 0.16536 | 1059.9420345548 |
| rs2823139 | A | G | 1.2497 | 0.33841 | 7.10068E-13 | 0.17411 | 51.5183069614462 |
| rs2834317 | A | G | 1.5431 | 0.15374 | 1.55812E-11 | 0.22886 | 45.4617164750477 |
| rs28374787 | G | A | 1.9408 | 0.077704 | 2.77172E-10 | 0.30751 | 39.832774190648 |
| rs28567372 | G | T | -0.79388 | 0.35448 | 4.00571E-06 | 0.17217 | 21.261418544586 |
| rs28617729 | T | C | 1.7794 | 0.16136 | 5.19039E-14 | 0.23639 | 56.6612883253565 |
| rs2871974 | T | C | -2.61 | 0.63986 | 1.39927E-52 | 0.171 | 232.962633831841 |
| rs2876454 | A | G | -1.3211 | 0.090333 | 3.75362E-06 | 0.28565 | 21.3894886479995 |
| rs28848873 | G | C | 1.0004 | 0.27767 | 8.87033E-08 | 0.18705 | 28.6041742414812 |
| rs2941484 | T | C | 2.5029 | 0.44541 | 2.00401E-51 | 0.16588 | 227.665077274239 |
| rs2943654 | T | C | 1.0753 | 0.64567 | 3.50308E-10 | 0.17137 | 39.3719723977566 |
| rs2973444 | C | T | -1.9684 | 0.085758 | 2.113E-11 | 0.29387 | 44.8656207175046 |

| | | | | | | | |
|---|---|---|---|---|---|---|---|
| rs3093662 | G | A | -1.5778 | 0.075465 | 3.5984E-07 | 0.31004 | 25.8979817705392 |
| rs3130672 | T | G | 1.1867 | 0.26914 | 1.33681E-10 | 0.18475 | 41.2582178251257 |
| rs323733 | A | T | -0.95026 | 0.75756 | 7.40167E-07 | 0.19195 | 24.507902169659 |
| rs33568 | G | T | 0.81468 | 0.50701 | 6.66653E-07 | 0.16389 | 24.709654490087 |
| rs33938520 | T | C | -1.0859 | 0.25276 | 9.61568E-09 | 0.18926 | 32.9199728651472 |
| rs34213329 | G | C | -19.814 | 0.022163 | 1E-200 | 0.55563 | 1271.65826487715 |
| rs34261493 | C | T | 1.1727 | 0.13617 | 9.77642E-07 | 0.23952 | 23.9710886558679 |
| rs345748 | G | A | 1.2324 | 0.75257 | 8.75588E-11 | 0.18997 | 42.0853368549098 |
| rs34650318 | G | C | -0.99227 | 0.17498 | 4.43568E-06 | 0.21619 | 21.0661898234372 |
| rs34811474 | A | G | -1.1488 | 0.23213 | 3.25979E-09 | 0.19412 | 35.0223885824767 |
| rs35016056 | G | T | 1.0854 | 0.54977 | 4.9204E-11 | 0.16512 | 43.2093515085327 |
| rs35342053 | T | C | 1.1694 | 0.12073 | 4.92845E-06 | 0.256 | 20.8662171271908 |
| rs354532 | T | C | 0.95926 | 0.53859 | 6.22515E-09 | 0.16508 | 33.7661250087263 |
| rs357488 | G | A | 1.4176 | 0.85247 | 9.54223E-10 | 0.23174 | 37.4199219784219 |
| rs358241 | G | A | 1.3993 | 0.85008 | 1.27711E-09 | 0.23051 | 36.8501789019266 |
| rs35859407 | A | G | -6.4714 | 0.0063893 | 5.36896E-10 | 1.0424 | 38.5411933494167 |
| rs36071802 | C | T | 1.9305 | 0.41557 | 3.89135E-30 | 0.16923 | 130.131482324013 |
| rs3746574 | C | T | -1.1894 | 0.51529 | 1.13214E-12 | 0.1672 | 50.6035279654513 |
| rs3755220 | T | C | 1.0721 | 0.18694 | 3.75267E-07 | 0.211 | 25.8168442816585 |
| rs3765154 | C | T | 0.86056 | 0.69838 | 1.6714E-06 | 0.17967 | 22.9408047932197 |
| rs3786900 | G | A | -1.0591 | 0.2684 | 1.1458E-08 | 0.18556 | 32.576369487577 |
| rs3824359 | C | T | 1.5345 | 0.14193 | 6.48634E-11 | 0.23491 | 42.6705508751688 |
| rs4001175 | A | C | 0.94364 | 0.29341 | 2.48691E-07 | 0.18292 | 26.6126285497153 |

| | | | | | | | |
|---|---|---|---|---|---|---|---|
| rs40270 | C | A | 1.7028 | 0.77305 | 4.51024E-17 | 0.20273 | 70.5486513724212 |
| rs405828 | T | C | -0.84666 | 0.60468 | 4.36496E-07 | 0.16758 | 25.5253194583739 |
| rs4077798 | G | A | -1.0484 | 0.78083 | 1.5361E-07 | 0.19977 | 27.5417136822593 |
| rs41264869 | T | C | 1.2771 | 0.17848 | 2.51142E-09 | 0.21425 | 35.5308520648188 |
| rs41290716 | A | C | 2.8066 | 0.070006 | 2.32113E-18 | 0.32108 | 76.4067992295502 |
| rs423144 | T | G | -2.9124 | 0.42619 | 2.93833E-69 | 0.16554 | 309.523492331033 |
| rs4285082 | T | C | -6.1473 | 0.978418 | 1.72068E-26 | 0.5771 | 113.465581001615 |
| rs4293567 | A | G | 1.6773 | 0.31451 | 1.85268E-21 | 0.17631 | 90.5034379219852 |
| rs429358 | C | T | -1.3887 | 0.15624 | 7.77088E-10 | 0.22581 | 37.8205642032619 |
| rs4326571 | A | G | -0.83884 | 0.71886 | 4.31559E-06 | 0.18253 | 21.1196749318018 |
| rs436780 | G | T | 1.0792 | 0.38414 | 1.74852E-10 | 0.1691 | 40.7299739154657 |
| rs4372964 | T | C | -0.88265 | 0.43396 | 1.0881E-07 | 0.16618 | 28.2109048175231 |
| rs4415952 | C | T | 1.1372 | 0.73315 | 8.86298E-10 | 0.18554 | 37.5660966337274 |
| rs4468717 | T | C | -1.7442 | 0.077674 | 1.21619E-08 | 0.30613 | 32.4622228746983 |
| rs45467396 | G | A | -2.7263 | 0.0198 | 3.56369E-06 | 0.58814 | 21.4873731826873 |
| rs45487598 | A | G | 1.4313 | 0.11689 | 2.45431E-08 | 0.25665 | 31.1011379212712 |
| rs455213 | C | T | 1.4598 | 0.4578 | 7.95243E-19 | 0.16474 | 78.5210447043295 |
| rs4575545 | A | G | -2.0883 | 0.30716 | 1.2627E-31 | 0.17846 | 136.930903419717 |
| rs4676084 | A | G | 0.77156 | 0.41765 | 3.76322E-06 | 0.16686 | 21.3812161594295 |
| rs4678419 | T | C | -0.76574 | 0.56883 | 4.26138E-06 | 0.16653 | 21.14341712 |
| rs4685262 | A | C | -1.1063 | 0.66092 | 3.19352E-10 | 0.17591 | 39.5514346588816 |
| rs4715208 | G | A | 0.9183 | 0.75523 | 1.46221E-06 | 0.19066 | 23.1978355338363 |

| | | | | | | | |
|---|---|---|---|---|---|---|---|
| rs4733363 | T | A | -1.0059 | 0.41167 | 2.19159E-09 | 0.16812 | 35.7987883285717 |
| rs4744712 | C | A | 1.0467 | 0.60157 | 4.21551E-10 | 0.16759 | 39.0072847124057 |
| rs4751640 | C | A | -1.0257 | 0.6938 | 1.0754E-08 | 0.17936 | 32.7029548182598 |
| rs4752992 | T | G | -1.2714 | 0.14233 | 5.89034E-08 | 0.23447 | 29.4027073369518 |
| rs478425 | T | G | -1.0729 | 0.36954 | 2.7359E-10 | 0.16995 | 39.854156931303 |
| rs4823085 | C | T | 1.2596 | 0.79874 | 7.05651E-10 | 0.20432 | 38.0050245939472 |
| rs4844981 | C | T | -0.92561 | 0.28681 | 3.30628E-07 | 0.18131 | 26.0621376313472 |
| rs4880352 | A | C | 0.86972 | 0.73029 | 2.65717E-06 | 0.18521 | 22.050961181065 |
| rs4915419 | A | C | -0.9274 | 0.58737 | 3.12493E-08 | 0.16757 | 30.629444300305 |
| rs4955640 | C | G | 0.87221 | 0.60517 | 2.05041E-07 | 0.1679 | 26.9859806587986 |
| rs4962687 | A | G | -1.3651 | 0.68142 | 9.84238E-15 | 0.17633 | 59.9341005487572 |
| rs508205 | A | G | 1.1419 | 0.55818 | 4.60787E-12 | 0.16508 | 47.8481002493537 |
| rs538656 | T | G | 1.6497 | 0.23401 | 1.58416E-17 | 0.1936 | 72.6100468002934 |
| rs541091 | A | G | -1.047 | 0.47483 | 1.90379E-10 | 0.16438 | 40.5688675585589 |
| rs541564 | A | G | 1.1063 | 0.35411 | 1.38609E-10 | 0.17239 | 41.1831118399939 |
| rs541928175 | T | C | 1.7812 | 0.065274 | 1.23911E-07 | 0.33685 | 27.9608096766386 |
| rs553653 | T | G | -0.96349 | 0.73933 | 2.46559E-07 | 0.18671 | 26.6291153507265 |
| rs55838345 | C | G | 1.3702 | 0.12404 | 4.20175E-08 | 0.24994 | 30.053417819846 |
| rs56379622 | A | G | -2.9742 | 0.0447 | 6.81397E-14 | 0.397 | 56.1250574904873 |
| rs569012321 | C | T | -1.1838 | 0.17835 | 4.7574E-08 | 0.21679 | 29.8178039080154 |
| rs572900800 | G | T | -8.7097 | 0.0074171 | 1.41026E-18 | 0.99006 | 77.3892854953092 |
| rs57675051 | T | C | 10.137 | 0.049186 | 4.85289E-144 | 0.3965 | 653.626999679333 |

| | | | | | | | |
|---|---|---|---|---|---|---|---|
| rs580241 | A | G | -1.2793 | 0.76343 | 9.15166E-11 | 0.1974 | 41.9998729762469 |
| rs58382609 | G | T | -0.82547 | 0.3551 | 1.48892E-06 | 0.17151 | 23.1644060263735 |
| rs603424 | A | G | 1.2044 | 0.168 | 3.96269E-08 | 0.21927 | 30.1703629165055 |
| rs60767324 | C | T | -1.5694 | 0.086706 | 7.83249E-08 | 0.2922 | 28.8472452754889 |
| rs61127279 | G | A | -0.85242 | 0.36555 | 6.11744E-07 | 0.17091 | 24.8753836280713 |
| rs6142206 | A | G | 1.2957 | 0.42115 | 6.27914E-15 | 0.16615 | 60.8142983117251 |
| rs61744628 | A | G | -2.3355 | 0.026939 | 3.97228E-06 | 0.50631 | 21.2776767516254 |
| rs61903695 | G | A | 0.86822 | 0.25598 | 3.88857E-06 | 0.18803 | 21.3207635331442 |
| rs62007715 | T | C | -0.99937 | 0.21451 | 6.12703E-07 | 0.20038 | 24.8737546256729 |
| rs62052820 | A | G | 2.112 | 0.22909 | 2.91072E-27 | 0.19526 | 116.99269129866 |
| rs62071306 | C | A | 1.3463 | 0.72846 | 4.01421E-13 | 0.18556 | 52.6395826950397 |
| rs62106258 | C | T | -2.8358 | 0.048343 | 1.20143E-13 | 0.38234 | 55.0109567848352 |
| rs62118698 | A | G | 0.89587 | 0.35203 | 3.2643E-07 | 0.1754 | 26.0872489690872 |
| rs62294340 | A | G | -1.4382 | 0.38339 | 1.57688E-17 | 0.16876 | 72.6268167304843 |
| rs62376957 | G | A | 1.0185 | 0.16657 | 3.93858E-06 | 0.22071 | 21.2948957823223 |
| rs62430713 | C | T | -1.172 | 0.17661 | 5.4606E-08 | 0.21561 | 29.5471024507901 |
| rs62435145 | T | G | 2.6861 | 0.69192 | 1.19371E-48 | 0.18322 | 214.929520557759 |
| rs62580785 | C | T | -1.108 | 0.17947 | 2.59753E-07 | 0.21512 | 26.5286662590032 |
| rs62618693 | T | C | -1.955 | 0.04536 | 6.64477E-07 | 0.39324 | 24.7158540746768 |
| rs6467228 | G | A | -1.1711 | 0.54649 | 1.32282E-12 | 0.16512 | 50.3020953233688 |
| rs653178 | T | C | -2.3031 | 0.51723 | 9.20874E-45 | 0.16405 | 197.092725427021 |
| rs6531977 | T | G | -1.5572 | 0.84157 | 4.57299E-12 | 0.22508 | 47.8643814542049 |

| | | | | | | | |
|---|---|---|---|---|---|---|---|
| rs6548237 | C | A | 1.0839 | 0.827 | 5.63365E-07 | 0.21663 | 25.0344876102837 |
| rs6589252 | C | A | -1.3421 | 0.82416 | 4.55837E-10 | 0.2153 | 38.8578857383149 |
| rs66492604 | C | T | 1.3449 | 0.1345 | 2.55558E-08 | 0.24146 | 31.0232339836723 |
| rs66704028 | T | C | -3.8935 | 0.35532 | 2.09411E-112 | 0.17275 | 507.974275742943 |
| rs67527161 | C | T | 1.0237 | 0.20772 | 4.57394E-07 | 0.20297 | 25.4377782411211 |
| rs67590677 | A | G | 1.6931 | 0.086283 | 7.54067E-09 | 0.29299 | 33.3931276570772 |
| rs676015 | C | T | -1.1571 | 0.6312 | 1.7555E-11 | 0.17206 | 45.2250883404904 |
| rs68046749 | G | A | -2.33 | 0.24067 | 5.76766E-31 | 0.20134 | 133.921155337146 |
| rs686364 | G | A | 1.4533 | 0.2346 | 5.51188E-14 | 0.19327 | 56.5430365204282 |
| rs6884239 | G | A | -0.81724 | 0.67463 | 3.18698E-06 | 0.17543 | 21.7014612310893 |
| rs6910439 | G | C | -0.95607 | 0.25522 | 3.78294E-07 | 0.18822 | 25.8015215411417 |
| rs6965823 | A | C | -1.0782 | 0.32213 | 7.45487E-10 | 0.17514 | 37.8987981727281 |
| rs700750 | A | C | 1.1329 | 0.62887 | 2.42605E-11 | 0.16964 | 44.5988951279216 |
| rs7093087 | A | G | 1.4131 | 0.17448 | 6.37382E-11 | 0.21624 | 42.7042600611207 |
| rs71321863 | A | G | 2.2514 | 0.029194 | 3.93867E-06 | 0.48787 | 21.2958299649428 |
| rs7145882 | C | T | -0.80937 | 0.65771 | 2.76229E-06 | 0.17265 | 21.976499329331 |
| rs7154553 | G | A | 1.4082 | 0.19838 | 8.24518E-12 | 0.20604 | 46.7114201699046 |
| rs7224610 | A | C | -2.2844 | 0.60224 | 1.27997E-41 | 0.169 | 182.712545271844 |
| rs7230932 | A | G | -0.84319 | 0.36862 | 7.35648E-07 | 0.17028 | 24.5200349787134 |
| rs72674300 | G | T | -4.0229 | 0.012941 | 3.08802E-07 | 0.78601 | 26.1950845980723 |
| rs72681698 | C | T | -5.8294 | 0.011039 | 1.08543E-13 | 0.78454 | 55.2096513845127 |

| | | | | | | | |
|---|---|---|---|---|---|---|---|
| rs72721542 | G | C | 1.0749 | 0.16695 | 1.06289E-06 | 0.22028 | 23.8113195850321 |
| rs727996 | T | G | 8.6718 | 0.036962 | 5.24566E-88 | 0.43592 | 395.733111852337 |
| rs72799820 | T | C | -1.3842 | 0.14999 | 1.78731E-09 | 0.23007 | 36.1972167740891 |
| rs72818964 | A | G | 1.2019 | 0.17609 | 2.29298E-08 | 0.21507 | 31.2301790362701 |
| rs72859654 | G | A | -1.0832 | 0.18981 | 2.4555E-07 | 0.20988 | 26.6362244275478 |
| rs72951456 | T | C | 2.146 | 0.048935 | 2.6171E-08 | 0.38559 | 30.9745770250852 |
| rs7302925 | G | A | -1.643 | 0.80212 | 1.28204E-15 | 0.20546 | 63.9466846448723 |
| rs7306544 | C | T | 1.257 | 0.10359 | 3.1268E-06 | 0.26961 | 21.7368213675784 |
| rs73153655 | G | A | 1.044 | 0.195 | 5.37193E-07 | 0.20827 | 25.1272544181584 |
| rs73205120 | C | T | 1.4374 | 0.077899 | 3.0128E-06 | 0.30779 | 21.8094012935236 |
| rs73796250 | A | G | 1.5385 | 0.0739 | 8.98649E-07 | 0.31316 | 24.1357003979386 |
| rs738408 | T | C | -1.5973 | 0.21583 | 1.00231E-15 | 0.19899 | 64.4329407352063 |
| rs742605 | G | A | 0.78618 | 0.44002 | 3.52834E-06 | 0.16953 | 21.5054399987405 |
| rs74606487 | G | A | -1.299 | 0.15062 | 3.00151E-08 | 0.23441 | 30.7088466869445 |
| rs7461961 | A | G | 0.93521 | 0.54612 | 1.87672E-08 | 0.16631 | 31.6212497771286 |
| rs74936120 | T | C | 2.7039 | 0.022411 | 1.41111E-06 | 0.56057 | 23.2658552372916 |
| rs7502296 | T | C | 0.8216 | 0.34098 | 2.323E-06 | 0.17395 | 22.3084537217166 |
| rs75212330 | T | A | 8.4394 | 0.008304 | 6.42244E-20 | 0.9236 | 83.4935195217512 |
| rs75294224 | A | G | -0.83749 | 0.39843 | 1.9269E-06 | 0.17591 | 22.6660413515022 |
| rs75588192 | A | G | 1.8763 | 0.13826 | 7.99466E-15 | 0.24154 | 60.3425844668924 |
| rs75622376 | T | C | -2.2223 | 0.034825 | 8.82165E-07 | 0.45203 | 24.1695368853309 |
| rs7565788 | C | T | -2.2628 | 0.75454 | 2.33346E-32 | 0.19105 | 140.279991181538 |
| rs75706763 | G | A | 1.7923 | 0.050904 | 2.30882E-06 | 0.37936 | 22.321111152619 |

| | | | | | | | |
|---|---|---|---|---|---|---|---|
| rs76158996 | A | T | -1.3203 | 0.134 | 1.39441E-07 | 0.25072 | 27.7309509163742 |
| rs7616014 | C | G | 1.1225 | 0.78126 | 1.95142E-08 | 0.19986 | 31.5441193326511 |
| rs76358556 | G | A | -1.7059 | 0.20379 | 5.21795E-17 | 0.20352 | 70.2571331334958 |
| rs7650788 | C | T | -0.90702 | 0.30651 | 4.44785E-07 | 0.17965 | 25.4904062864812 |
| rs76768110 | A | G | -1.2107 | 0.14438 | 2.509E-07 | 0.23476 | 26.5963694413097 |
| rs76895963 | G | T | -4.3713 | 0.021069 | 3.82208E-12 | 0.62952 | 48.2169388710032 |
| rs7696556 | C | A | -1.4258 | 0.1395 | 1.7916E-09 | 0.23701 | 36.1893975997466 |
| rs7728485 | G | A | 0.87449 | 0.27792 | 1.86449E-06 | 0.18342 | 22.730751323142 |
| rs7736102 | A | G | 1.1526 | 0.56872 | 3.54079E-12 | 0.16573 | 48.3673667196789 |
| rs77408475 | T | C | 1.3102 | 0.10125 | 2.19392E-06 | 0.27672 | 22.4177264795458 |
| rs77542162 | G | A | -4.1928 | 0.022979 | 1.95164E-14 | 0.54778 | 58.5859428826305 |
| rs77665054 | A | G | -2.1806 | 0.039026 | 3.059E-07 | 0.42591 | 26.2128242214253 |
| rs7828510 | C | T | -2.4314 | 0.027354 | 2.81022E-06 | 0.51906 | 21.9419476083574 |
| rs784257 | C | T | 1.1765 | 0.81295 | 2.5784E-08 | 0.2113 | 31.0014739567673 |
| rs78565962 | T | A | 1.5952 | 0.06691 | 1.26949E-06 | 0.32929 | 23.4677077702526 |
| rs78653058 | T | G | -3.0049 | 0.020177 | 9.16094E-07 | 0.61214 | 24.0966130025088 |
| rs78671965 | T | A | 2.1429 | 0.055477 | 3.50397E-09 | 0.36283 | 34.8814801940031 |
| rs78852738 | C | A | -2.2361 | 0.029424 | 4.06912E-06 | 0.48528 | 21.2322065835732 |
| rs79061817 | A | G | 0.91097 | 0.23574 | 2.70639E-06 | 0.19415 | 22.0156156237046 |
| rs79099587 | T | A | 1.3562 | 0.086382 | 3.55468E-06 | 0.29254 | 21.4918816217781 |
| rs79239275 | A | C | 2.1468 | 0.045961 | 4.29339E-08 | 0.39186 | 30.0136475807668 |
| rs79240721 | T | C | -2.4442 | 0.042692 | 3.34549E-09 | 0.41331 | 34.9718918841041 |
| rs79295634 | G | A | 1.5604 | 0.06672 | 2.11861E-06 | 0.32906 | 22.486360572128 |

| | | | | | | | |
|---|---|---|---|---|---|---|---|
| rs79411582 | C | T | -10.147 | 0.0093515 | 6.86278E-30 | 0.89335 | 129.011822707345 |
| rs8024386 | C | A | 1.2247 | 0.22877 | 6.2004E-10 | 0.198 | 38.2583758175045 |
| rs8025378 | C | T | 0.86422 | 0.26112 | 3.78242E-06 | 0.18694 | 21.3718256414003 |
| rs80271085 | T | C | 1.7663 | 0.048288 | 3.91571E-06 | 0.38265 | 21.307032894714 |
| rs8048364 | G | T | -1.2842 | 0.16352 | 6.70147E-09 | 0.22147 | 33.6227315924877 |
| rs807624 | T | G | -1.4797 | 0.35776 | 4.76321E-18 | 0.17088 | 74.982920334575 |
| rs8079951 | A | T | -1.1022 | 0.81626 | 2.02591E-07 | 0.21208 | 27.009643858987 |
| rs8110079 | T | G | -0.93646 | 0.75493 | 1.89601E-06 | 0.19656 | 22.6978985275208 |
| rs8192362 | A | G | 1.3 | 0.72801 | 1.86982E-12 | 0.18455 | 49.6199222425482 |
| rs833805 | G | A | 2.0666 | 0.8848 | 1.68229E-14 | 0.26933 | 58.8763477467704 |
| rs836968 | T | C | -1.2114 | 0.27078 | 7.28618E-11 | 0.18595 | 42.4404760887445 |
| rs854917 | T | C | -1.0558 | 0.73758 | 1.75999E-08 | 0.18738 | 31.7478500152089 |
| rs871375 | A | G | -1.4088 | 0.65649 | 3.39469E-16 | 0.17267 | 66.56752066 |
| rs881795 | T | C | 2.4539 | 0.028623 | 6.67099E-07 | 0.49366 | 24.7090084151438 |
| rs894680 | A | G | -0.81726 | 0.38624 | 1.2305E-06 | 0.16848 | 23.529963460774 |
| rs9297949 | C | A | 1.6064 | 0.52978 | 1.30617E-22 | 0.16416 | 95.7569220393113 |
| rs9333592 | T | C | -1.9156 | 0.078094 | 7.19996E-10 | 0.31089 | 37.9658934712206 |
| rs9534949 | G | C | -1.2221 | 0.72989 | 3.3335E-11 | 0.1843 | 43.9703873598244 |
| rs9599875 | T | A | -1.3138 | 0.30319 | 1.92752E-13 | 0.17864 | 54.0877068071126 |
| rs9607693 | A | G | -0.78064 | 0.57623 | 4.02856E-06 | 0.16934 | 21.2510290262629 |
| rs963837 | C | T | -2.0336 | 0.45294 | 4.40859E-35 | 0.16454 | 152.751524848785 |
| rs9660710 | C | A | 1.8548 | 0.933366 | 1.67101E-08 | 0.32869 | 31.8433437785387 |

| | | | | | | | |
|---|---|---|---|---|---|---|---|
| rs9672549 | C | T | 0.85702 | 0.62134 | 5.37836E-07 | 0.17098 | 25.123998707744 |
| rs9704692 | T | C | 0.97142 | 0.46448 | 5.76806E-09 | 0.16681 | 33.9130921859445 |
| rs9807214 | A | G | 1.0267 | 0.30265 | 9.1865E-09 | 0.17871 | 33.0055434683754 |
| rs9844972 | C | G | 1.5129 | 0.071303 | 3.09963E-06 | 0.32436 | 21.7552128178649 |
| rs9849171 | C | G | -0.92773 | 0.3599 | 5.69049E-08 | 0.1709 | 29.4683995459481 |
| rs9880232 | A | C | -1.1683 | 0.70198 | 8.89201E-11 | 0.18016 | 42.0522735527861 |
| rs9889162 | A | T | -0.92786 | 0.27045 | 4.93549E-07 | 0.1845 | 25.2912117813307 |
| rs9891685 | A | G | 0.88472 | 0.26831 | 2.07788E-06 | 0.18642 | 22.5228921197157 |
| rs9932625 | A | G | 1.5258 | 0.22886 | 6.09256E-15 | 0.19555 | 60.8803379222137 |

**8.TBIL-Keratoconus**

| | | | | | | | |
|---|---|---|---|---|---|---|---|
| rs10023050 | G | A | 0.067044 | 0.39404 | 2.5672E-10 | 0.010603 | 39.9815639578838 |
| rs1012384 | A | G | 0.070286 | 0.82658 | 2.83949E-07 | 0.013691 | 26.3551068828968 |
| rs102275 | C | T | -0.071055 | 0.35172 | 5.93472E-11 | 0.010855 | 42.8476589350981 |
| rs1047891 | A | C | 0.08994 | 0.31574 | 7.12853E-16 | 0.011147 | 65.1009356777239 |
| rs10495928 | G | A | -0.054546 | 0.33575 | 6.68205E-07 | 0.010974 | 24.7054842557287 |
| rs10733608 | T | G | -0.053278 | 0.48558 | 3.45327E-07 | 0.010453 | 25.9783397701634 |
| rs10769315 | C | T | 0.058682 | 0.78907 | 3.8952E-06 | 0.01271 | 21.3165137867479 |
| rs10876376 | A | G | 0.057242 | 0.5479 | 4.49252E-08 | 0.010464 | 29.9248238815604 |
| rs10881578 | G | A | -0.059565 | 0.28456 | 2.16521E-07 | 0.011489 | 26.8791356949157 |
| rs11121 | T | C | -0.052818 | 0.49809 | 3.56607E-07 | 0.010375 | 25.9170257435259 |
| rs11124287 | A | G | 0.060233 | 0.66301 | 4.36898E-08 | 0.011001 | 29.9779634600985 |
| rs11217176 | A | G | 0.054286 | 0.27048 | 3.37233E-06 | 0.011682 | 21.5942809446895 |

| | | | | | | | |
|---|---|---|---|---|---|---|---|
| rs11224309 | G | C | 0.093028 | 0.097333 | 1.04381E-07 | 0.01749 | 28.2907991758462 |
| rs113117433 | T | C | -0.25348 | 0.020235 | 4.11244E-11 | 0.038404 | 43.5644204903494 |
| rs114165349 | C | G | 0.26919 | 0.023542 | 5.17726E-15 | 0.034411 | 61.1956742428589 |
| rs11495610 | T | C | -0.062958 | 0.21323 | 8.65765E-07 | 0.012796 | 24.207550697829 |
| rs11635675 | G | T | 0.10214 | 0.34308 | 7.82889E-21 | 0.010909 | 87.6635976742264 |
| rs11656978 | A | G | -0.062145 | 0.22969 | 4.86575E-07 | 0.012351 | 25.3166132809696 |
| rs11727331 | A | G | 0.12692 | 0.051756 | 8.06826E-08 | 0.023654 | 28.7904470349802 |
| rs11744385 | T | C | -0.095455 | 0.066892 | 4.25726E-06 | 0.020758 | 21.1457851422423 |
| rs1231204 | C | G | -0.11143 | 0.950556 | 3.12838E-06 | 0.0239 | 21.7373163348429 |
| rs12414160 | G | A | -0.16734 | 0.087308 | 7.7589E-20 | 0.018355 | 83.1166858403606 |
| rs12673943 | C | T | 0.049656 | 0.36565 | 4.44467E-06 | 0.01082 | 21.0613599060266 |
| rs12814270 | G | A | 0.27958 | 0.22095 | 1.95884E-110 | 0.012517 | 498.89501336875 |
| rs1292043 | G | A | -0.089185 | 0.20213 | 4.53524E-12 | 0.012889 | 47.8787333348972 |
| rs13092376 | C | A | 0.049805 | 0.41903 | 2.29208E-06 | 0.010539 | 22.3328704292926 |
| rs13201752 | G | A | -0.069985 | 0.31854 | 2.98552E-10 | 0.011109 | 39.6878403243088 |
| rs1392436 | G | A | 0.061869 | 0.33062 | 1.9762E-08 | 0.01102 | 31.5195835199017 |
| rs143663553 | G | C | 2.513 | 0.014563 | 1E-200 | 0.045799 | 3010.72058956035 |
| rs1474865 | C | G | -0.10115 | 0.11746 | 4.74974E-10 | 0.016244 | 38.7742386482362 |
| rs149767042 | A | G | 2.3396 | 0.027979 | 1E-200 | 0.031325 | 5578.25717156824 |
| rs1519818 | A | G | 0.050529 | 0.60857 | 2.0281E-06 | 0.010636 | 22.5695144421091 |
| rs1688264 | G | T | 0.063553 | 0.53418 | 1.1375E-09 | 0.010437 | 37.0781632019358 |
| rs16947051 | C | G | -0.052902 | 0.337 | 1.38839E-06 | 0.01096 | 23.2981019014662 |
| rs17316633 | A | G | 0.074281 | 0.26445 | 2.58178E-10 | 0.011749 | 39.9715640651964 |

| | | | | | | | |
|---|---|---|---|---|---|---|---|
| rs17476364 | C | T | 0.30981 | 0.10984 | 6.45803E-78 | 0.016576 | 349.324345279273 |
| rs1762486 | A | G | 0.074524 | 0.65639 | 1.57761E-11 | 0.011056 | 45.4353335526931 |
| rs17862876 | T | G | 2.4456 | 0.017369 | 1E-200 | 0.039366 | 3859.45286711873 |
| rs17869072 | C | T | 0.29196 | 0.14866 | 7.26775E-89 | 0.014604 | 399.669058835283 |
| rs1800562 | A | G | 0.22095 | 0.078833 | 1.80385E-30 | 0.019256 | 131.659840207385 |
| rs1800961 | T | C | 0.26213 | 0.031428 | 1.07201E-18 | 0.029694 | 77.9279968925876 |
| rs1807609 | C | T | 0.068569 | 0.69866 | 1.40991E-09 | 0.011325 | 36.6586676578118 |
| rs1810504 | C | G | 0.071082 | 0.8462 | 7.46466E-07 | 0.014363 | 24.4921225106881 |
| rs181207 | T | C | -0.068298 | 0.3362 | 5.53911E-10 | 0.01101 | 38.4803311620896 |
| rs1848984 | T | A | 0.14173 | 0.18798 | 9.72971E-27 | 0.01324 | 114.589564910885 |
| rs1874121 | T | C | 0.085054 | 0.33965 | 8.87769E-15 | 0.010968 | 60.135658161714 |
| rs1896995 | T | C | -0.13685 | 0.485 | 2.41379E-39 | 0.010427 | 172.253606200962 |
| rs190591485 | C | T | 2.3287 | 0.036475 | 1E-200 | 0.030129 | 5973.86106498158 |
| rs2010127 | C | T | -0.064651 | 0.82858 | 2.71738E-06 | 0.013781 | 22.0082998559188 |
| rs2061298 | G | A | 0.051319 | 0.32662 | 3.74171E-06 | 0.011095 | 21.3943562659432 |
| rs2068888 | A | G | 0.12072 | 0.44882 | 4.27464E-31 | 0.010409 | 134.504837107979 |
| rs2087826 | A | G | 0.049707 | 0.53541 | 1.6966E-06 | 0.010384 | 22.9141215656557 |
| rs2235567 | A | G | -0.05502 | 0.36049 | 3.66514E-07 | 0.010819 | 25.8621363416971 |
| rs2321536 | T | C | -0.057829 | 0.42649 | 1.6838E-07 | 0.011054 | 27.3684316402432 |
| rs2522051 | C | T | -0.060983 | 0.45369 | 4.62967E-09 | 0.010407 | 34.3371248667045 |
| rs2535613 | G | A | -0.059498 | 0.6299 | 1.12279E-07 | 0.011214 | 28.1501749039434 |
| rs2587534 | A | G | 0.07894 | 0.51796 | 3.48498E-14 | 0.010415 | 57.4477682785768 |
| rs2657878 | T | C | 0.064001 | 0.17899 | 2.42242E-06 | 0.013575 | 22.227535341594 |

| | | | | | | | |
|---|---|---|---|---|---|---|---|
| rs2670430 | G | C | 0.11171 | 0.87787 | 1.74221E-12 | 0.015836 | 49.7611700911774 |
| rs2792703 | T | C | -0.073408 | 0.72134 | 2.18072E-10 | 0.011563 | 40.3035405080285 |
| rs28910284 | A | G | 0.056221 | 0.26559 | 1.66752E-06 | 0.011737 | 22.9445946401636 |
| rs2968478 | G | T | -0.072259 | 0.58136 | 1.42856E-11 | 0.010697 | 45.6307249752405 |
| rs340005 | A | G | -0.09449 | 0.62059 | 8.40814E-19 | 0.010671 | 78.4077379235897 |
| rs34265667 | A | G | 0.25461 | 0.03472 | 2.46604E-19 | 0.028319 | 80.8337176826432 |
| rs34473081 | A | G | 0.049853 | 0.57381 | 2.47349E-06 | 0.010584 | 22.186071073107 |
| rs34755157 | T | C | -0.072606 | 0.17025 | 1.64869E-07 | 0.013869 | 27.4064126658365 |
| rs35115456 | T | G | -0.12798 | 0.052182 | 7.03655E-08 | 0.023742 | 29.0567526738113 |
| rs372338580 | T | C | 0.10669 | 0.05714 | 2.24621E-06 | 0.022556 | 22.3728198085103 |
| rs3735964 | A | C | -0.081675 | 0.10639 | 1.17649E-06 | 0.016807 | 23.6154025260203 |
| rs3768321 | T | G | -0.069753 | 0.19677 | 9.24932E-08 | 0.01306 | 28.5257383445094 |
| rs3851298 | C | T | -0.082347 | 0.901968 | 2.3871E-06 | 0.017455 | 22.2563183087198 |
| rs4149056 | C | T | 0.62359 | 0.15089 | 1E-200 | 0.014427 | 1868.28740965634 |
| rs4410790 | C | T | -0.12041 | 0.63423 | 5.05126E-29 | 0.010769 | 125.017792264046 |
| rs4475971 | A | G | -0.08689 | 0.56084 | 1.35021E-16 | 0.010507 | 68.3879517057027 |
| rs450244 | C | T | -0.21226 | 0.910761 | 2.67855E-31 | 0.018239 | 135.435258037356 |
| rs4695913 | A | C | -0.053351 | 0.53189 | 3.17592E-07 | 0.010435 | 26.1395247881506 |
| rs4791212 | T | C | -0.078223 | 0.20418 | 1.2458E-09 | 0.012878 | 36.8951738251842 |
| rs4858819 | T | C | 0.060353 | 0.26348 | 3.07929E-07 | 0.011791 | 26.1995548888489 |
| rs4874171 | T | C | -0.053571 | 0.63159 | 6.84857E-07 | 0.010788 | 24.6589793843069 |
| rs4919820 | G | T | 0.066318 | 0.84371 | 3.33388E-06 | 0.014265 | 21.6130846896653 |

| | | | | | | | |
|---|---|---|---|---|---|---|---|
| rs4925546 | G | A | -0.066518 | 0.63035 | 6.00814E-10 | 0.010745 | 38.3233082445394 |
| rs4945881 | A | G | -0.063457 | 0.18464 | 2.05779E-06 | 0.013366 | 22.5399851621483 |
| rs499974 | A | C | 0.11364 | 0.15608 | 1.71356E-15 | 0.014275 | 63.373498483246 |
| rs5023360 | A | T | -0.086621 | 0.10885 | 2.01999E-07 | 0.016666 | 27.0135149654905 |
| rs536899661 | A | C | 0.13402 | 0.047143 | 1.46619E-07 | 0.025494 | 27.6350802868152 |
| rs55649657 | G | C | 0.091747 | 0.2203 | 2.28455E-13 | 0.012514 | 53.7512922577429 |
| rs55781538 | C | T | 2.4919 | 0.022295 | 1E-200 | 0.035572 | 4907.29446999823 |
| rs55889152 | T | C | 0.082176 | 0.10212 | 1.55561E-06 | 0.017106 | 23.077593884684 |
| rs55974805 | T | C | 0.058711 | 0.24537 | 1.083E-06 | 0.012041 | 23.7744954410855 |
| rs56389940 | A | C | -0.057734 | 0.32428 | 1.8647E-07 | 0.011076 | 27.170327171982 |
| rs59616136 | A | G | 0.13158 | 0.092856 | 5.27594E-13 | 0.018229 | 52.101657620463 |
| rs6000553 | G | A | 0.09098 | 0.53287 | 2.48028E-18 | 0.010417 | 76.2788264959703 |
| rs61061000 | T | C | 0.082793 | 0.1108 | 5.84655E-07 | 0.016571 | 24.9624536108009 |
| rs6129760 | G | A | 0.06559 | 0.32286 | 3.37684E-09 | 0.011094 | 34.953961626535 |
| rs61984409 | C | A | -0.050682 | 0.3615 | 2.59119E-06 | 0.010781 | 22.0997246905218 |
| rs62020701 | C | G | -0.085051 | 0.098316 | 1.0847E-06 | 0.017444 | 23.7719144644544 |
| rs62088207 | T | C | 0.068011 | 0.15745 | 1.87021E-06 | 0.014267 | 22.7242974320544 |
| rs62192912 | T | C | -0.713 | 0.3053 | 1E-200 | 0.011407 | 3906.91212416911 |
| rs62396718 | C | T | -0.079482 | 0.10479 | 3.27899E-06 | 0.017083 | 21.6474506385931 |
| rs635634 | T | C | -0.090527 | 0.18392 | 1.63983E-11 | 0.013441 | 45.3618294091977 |
| rs662138 | G | C | -0.098706 | 0.18743 | 9.89464E-14 | 0.013262 | 55.3944844430995 |
| rs6750065 | T | C | 0.083615 | 0.73459 | 1.44112E-12 | 0.011809 | 50.1348372431425 |
| rs6750559 | A | G | -0.07904 | 0.38959 | 9.95405E-14 | 0.010621 | 55.3809640982323 |

| | | | | | | | |
|---|---|---|---|---|---|---|---|
| rs6762415 | G | T | -0.050622 | 0.53489 | 1.1623E-06 | 0.010412 | 23.6378377053394 |
| rs6802898 | T | C | -0.088014 | 0.12224 | 2.48731E-08 | 0.015789 | 31.0736117293416 |
| rs6822348 | T | A | -0.066919 | 0.69842 | 3.21647E-09 | 0.011303 | 35.0516846786171 |
| rs687339 | T | C | -0.19337 | 0.77192 | 3.92374E-55 | 0.012362 | 244.679836907931 |
| rs6926614 | A | G | -0.069998 | 0.80824 | 1.31051E-07 | 0.013264 | 27.849647516947 |
| rs6943025 | A | T | 0.086979 | 0.85798 | 7.29508E-09 | 0.015038 | 33.4538515911736 |
| rs7047279 | C | T | 0.055414 | 0.56764 | 1.3498E-07 | 0.010511 | 27.7938298801876 |
| rs7139079 | A | G | 0.071223 | 0.5949 | 1.685E-11 | 0.010581 | 45.3090089710791 |
| rs719928 | A | G | 0.09489 | 0.069958 | 4.06584E-06 | 0.020592 | 21.2344638797015 |
| rs7205582 | A | G | 0.050693 | 0.39119 | 1.87478E-06 | 0.010635 | 22.7205303596308 |
| rs7213285 | A | G | 0.066342 | 0.16421 | 2.23939E-06 | 0.014024 | 22.3784900639636 |
| rs7222046 | G | A | 0.074576 | 0.4314 | 1.40929E-12 | 0.010528 | 50.1769067656687 |
| rs72681698 | C | T | 0.27171 | 0.011023 | 4.33192E-08 | 0.049611 | 29.9952681715962 |
| rs72729610 | G | A | -0.067298 | 0.16715 | 1.27421E-06 | 0.013893 | 23.4644138902229 |
| rs72835688 | T | C | -0.10344 | 0.059428 | 4.89892E-06 | 0.022639 | 20.876611588507 |
| rs72844048 | A | G | 0.18049 | 0.020804 | 3.38376E-06 | 0.038848 | 21.5857143769281 |
| rs7312591 | C | G | -0.19238 | 0.927273 | 6.99359E-22 | 0.02001 | 92.4321659515946 |
| rs73169738 | G | A | 0.10188 | 0.077508 | 1.59089E-07 | 0.019436 | 27.4765093260785 |
| rs738409 | G | C | 0.063153 | 0.21587 | 5.07645E-07 | 0.012572 | 25.2334535385984 |
| rs73971708 | G | A | 0.067166 | 0.17109 | 1.09979E-06 | 0.013783 | 23.7470094098844 |
| rs75130623 | T | C | -1.3251 | 0.018819 | 1E-200 | 0.041634 | 1012.97446681839 |
| rs7526446 | A | C | 0.069682 | 0.63709 | 1.62761E-10 | 0.010899 | 40.8757162460238 |
| rs76083814 | T | C | 0.084672 | 0.093699 | 2.063E-06 | 0.017836 | 22.5362771731232 |

| | | | | | | | |
|---|---|---|---|---|---|---|---|
| rs76384978 | G | A | 0.5002 | 0.013708 | 2.93089E-29 | 0.04454 | 126.120131760474 |
| rs76895963 | G | T | 0.22519 | 0.021083 | 1.49751E-08 | 0.039771 | 32.0599356438541 |
| rs7770144 | A | G | -0.053956 | 0.45619 | 2.21529E-07 | 0.010415 | 26.8385105248337 |
| rs78480755 | G | A | -0.12482 | 0.040685 | 1.9593E-06 | 0.026235 | 22.6362085153029 |
| rs7907226 | C | G | -0.097065 | 0.15378 | 1.35425E-11 | 0.014353 | 45.7338132945311 |
| rs79260437 | T | C | -0.39614 | 0.0098037 | 5.15703E-14 | 0.052621 | 56.673060547268 |
| rs7947951 | G | A | -0.09012 | 0.69118 | 8.93511E-16 | 0.011207 | 64.6637800604926 |
| rs79485702 | G | A | 0.06166 | 0.28916 | 1.4773E-07 | 0.011733 | 27.6176273843426 |
| rs8041523 | T | C | 0.052276 | 0.42586 | 6.14257E-07 | 0.010483 | 24.8674351121995 |
| rs857720 | C | T | -0.11102 | 0.26657 | 2.47742E-21 | 0.011707 | 89.9308453255882 |
| rs9410397 | C | T | 0.18673 | 0.056588 | 1.60066E-16 | 0.022636 | 68.0497118071412 |
| rs9438901 | A | G | 0.086254 | 0.85781 | 6.06443E-09 | 0.014833 | 33.8141100603009 |
| rs9616 | T | A | 0.057305 | 0.29558 | 4.64954E-07 | 0.011369 | 25.4060978938995 |
| rs964184 | C | G | -0.13051 | 0.86793 | 1.38835E-17 | 0.015289 | 72.8663280049291 |
| rs964653 | A | T | -0.10735 | 0.079726 | 2.0659E-08 | 0.019148 | 31.4307487013875 |
| rs9826148 | T | C | -0.086051 | 0.10871 | 2.62367E-07 | 0.016713 | 26.509435712484 |
| rs9923299 | T | C | 0.048141 | 0.46312 | 3.72229E-06 | 0.010406 | 21.4022798244275 |
| rs9971862 | G | T | -0.061931 | 0.22238 | 7.25254E-07 | 0.0125 | 24.5467288685584 |
| **9.Retinol-Keratoconus** | | | | | | | |
| rs117219913 | C | T | 0.0476864 | 0.07834 | 4.39997E-06 | 0.0103813 | 21.0994824409391 |
| rs117669768 | A | G | 0.0789545 | 0.038236 | 8.99995E-08 | 0.0147682 | 28.5814893384142 |
| rs149577802 | T | C | -0.108795 | 0.015116 | 3.40001E-06 | 0.0234115 | 21.5946218364097 |
| rs2126371 | T | C | -0.0289453 | 0.317531 | 1.29999E-06 | 0.00597696 | 23.4520937789043 |

| | | | | | | | |
|---|---|---|---|---|---|---|---|
| rs3213829 | G | T | 0.025976 | 0.546482 | 3.59998E-06 | 0.00560984 | 21.4402483853252 |
| rs692790 | C | T | 0.0404149 | 0.874331 | 1.40001E-06 | 0.00838208 | 23.2469309041744 |
| rs74977546 | A | G | -0.063514 | 0.052896 | 5.49997E-07 | 0.0126784 | 25.095522426514 |
| rs909570 | A | G | -0.053135 | 0.938952 | 4.39997E-06 | 0.0115717 | 21.08401171 |

**10.Albumin-Keratoconus**

| | | | | | | | |
|---|---|---|---|---|---|---|---|
| rs10069690 | T | C | 0.0256734 | 0.258948 | 5.19996E-08 | 0.00471521 | 29.64590383 |
| rs10419198 | T | C | -0.0559249 | 0.253459 | 2.70023E-32 | 0.0047272 | 139.959531 |
| rs10518679 | C | T | 0.0268431 | 0.151199 | 3.09999E-06 | 0.00575268 | 21.77333254 |
| rs10796927 | C | T | 0.0241302 | 0.284864 | 1.5E-07 | 0.00459843 | 27.53611606 |
| rs10828249 | A | G | -0.0216302 | 0.343064 | 6.49995E-07 | 0.00434783 | 24.75004315 |
| rs10898 | T | C | 0.0219704 | 0.736198 | 3.89996E-06 | 0.00475738 | 21.32751435 |
| rs11048591 | C | A | 0.0212942 | 0.336274 | 1.2E-06 | 0.00438373 | 23.59581935 |
| rs11121396 | C | T | 0.0201616 | 0.413112 | 1.79999E-06 | 0.00422209 | 22.80315898 |
| rs11128594 | G | A | -0.0359885 | 0.193633 | 6.20012E-12 | 0.00523496 | 47.26076597 |
| rs112026770 | A | G | -0.0312658 | 0.147834 | 8.50002E-08 | 0.00583625 | 28.69930086 |
| rs114949263 | C | T | 0.0499753 | 0.111367 | 3.29989E-14 | 0.00658947 | 57.51880432 |
| rs115744844 | C | A | 0.0917847 | 0.015915 | 1.79999E-07 | 0.0175838 | 27.24677558 |
| rs11589479 | A | G | 0.0349953 | 0.166956 | 2.69998E-10 | 0.00554322 | 39.85613775 |
| rs116446940 | C | T | -0.107534 | 0.009199 | 2.59998E-06 | 0.0228622 | 22.123588 |
| rs12433544 | A | T | 0.0209287 | 0.627853 | 1E-06 | 0.00428646 | 23.83894077 |
| rs1260326 | C | T | -0.0444079 | 0.603972 | 7.39946E-26 | 0.00422344 | 110.5573996 |

| | | | | | | | |
|---|---|---|---|---|---|---|---|
| rs12613605 | T | G | 0.0231752 | 0.212963 | 4.09996E-06 | 0.00503022 | 21.22623765 |
| rs13079232 | G | C | -0.0233995 | 0.247145 | 1.09999E-06 | 0.00480045 | 23.76015415 |
| rs13108218 | G | A | -0.0281529 | 0.615358 | 4.60045E-11 | 0.00427562 | 43.35588699 |
| rs137957801 | G | A | -0.0366136 | 0.083513 | 1.09999E-06 | 0.00750063 | 23.82809813 |
| rs139974673 | C | T | 0.120695 | 0.025922 | 1.9002E-20 | 0.0130233 | 85.8887868 |
| rs140567911 | C | T | -0.053586 | 0.038583 | 7.90005E-07 | 0.0108497 | 24.39311256 |
| rs1455590 | T | C | 0.0230297 | 0.23346 | 2.99999E-06 | 0.00493391 | 21.78683352 |
| rs1461729 | G | A | 0.0603088 | 0.899226 | 1.69981E-18 | 0.0068704 | 77.05437972 |
| rs150687233 | T | C | 0.0199376 | 0.353543 | 4.60002E-06 | 0.00435002 | 21.00696201 |
| rs17580 | A | T | 0.145105 | 0.047866 | 7.19946E-51 | 0.00967283 | 225.0389251 |
| rs1791936 | A | G | 0.0228082 | 0.603746 | 6.4E-08 | 0.00421854 | 29.23194294 |
| rs186663077 | T | C | 0.0854356 | 0.015431 | 7.00003E-07 | 0.0172197 | 24.61651593 |
| rs190366150 | T | C | -0.0828637 | 0.016549 | 1.7E-06 | 0.0172994 | 22.94386409 |
| rs1962003 | A | C | -0.0224076 | 0.701712 | 7.49998E-07 | 0.0045292 | 24.4764083 |
| rs2112469 | A | G | 0.019141 | 0.54359 | 4.20001E-06 | 0.00415974 | 21.17370577 |
| rs2116327 | A | G | 0.0220371 | 0.326139 | 5.60003E-07 | 0.00440409 | 25.03782006 |
| rs2316307 | C | T | -0.0210809 | 0.621957 | 8E-07 | 0.00427239 | 24.34649886 |
| rs2556569 | T | C | -0.048338 | 0.049261 | 1.09999E-06 | 0.00991465 | 23.76963864 |
| rs2691584 | C | T | 0.0222855 | 0.413504 | 1.09999E-07 | 0.00419618 | 28.20567814 |
| rs28929474 | T | C | 0.428915 | 0.019977 | 7.29458E-185 | 0.0147921 | 840.7808297 |
| rs2933243 | A | G | 0.0303081 | 0.18319 | 1.40001E-08 | 0.0053382 | 32.23500494 |

| | | | | | | | |
|---|---|---|---|---|---|---|---|
| rs34284056 | A | C | 0.026938 | 0.274775 | 6.49995E-09 | 0.00464163 | 33.68136318 |
| rs34754216 | T | C | -0.0302553 | 0.424071 | 1.80011E-12 | 0.0042924 | 49.68239859 |
| rs3740688 | T | G | 0.0321874 | 0.545016 | 8.49963E-15 | 0.00414786 | 60.21762395 |
| rs3749748 | T | C | -0.023417 | 0.246388 | 1.2E-06 | 0.00481436 | 23.65840095 |
| rs3768321 | T | G | -0.0314995 | 0.196531 | 1.5E-09 | 0.00520982 | 36.55627057 |
| rs3818871 | A | G | -0.0215098 | 0.277451 | 3.09999E-06 | 0.00461182 | 21.75344519 |
| rs4470390 | G | A | -0.0255899 | 0.768302 | 1.89998E-07 | 0.00490821 | 27.18259444 |
| rs4886992 | C | T | -0.0316658 | 0.206119 | 5.69994E-10 | 0.00510933 | 38.41077073 |
| rs555754 | A | G | -0.0205267 | 0.468421 | 7.39997E-07 | 0.00414669 | 24.50389548 |
| rs55881006 | A | G | -0.0695923 | 0.033778 | 1.2E-09 | 0.0114547 | 36.91092604 |
| rs56347111 | A | C | 0.0227383 | 0.23376 | 3.2E-06 | 0.00488513 | 21.66525143 |
| rs565840574 | G | A | 0.0271702 | 0.351588 | 3.09999E-07 | 0.00531181 | 26.16378708 |
| rs57274629 | G | A | 0.0286584 | 0.357884 | 4.60045E-11 | 0.00435395 | 43.32484377 |
| rs58087925 | T | C | -0.0229299 | 0.239337 | 2.69998E-06 | 0.00488281 | 22.05284732 |
| rs58546652 | T | C | -0.0253582 | 0.627861 | 3.09999E-09 | 0.00427752 | 35.14412605 |
| rs58895965 | A | C | 0.0633893 | 0.174684 | 3.50026E-31 | 0.00545847 | 134.862143 |
| rs59916403 | T | G | -0.0211051 | 0.351564 | 1.09999E-06 | 0.00433104 | 23.74599866 |
| rs607087 | T | C | -0.0218047 | 0.291788 | 1.6E-06 | 0.00454266 | 23.0398563 |
| rs61653336 | A | G | -0.0369604 | 0.165279 | 3.19963E-11 | 0.00556946 | 44.03998344 |
| rs631695 | G | T | 0.019736 | 0.582075 | 2.39999E-06 | 0.00418738 | 22.2143456 |
| rs633683 | C | T | 0.0207685 | 0.597775 | 8.60003E-07 | 0.00422081 | 24.2113305 |

| | | | | | | | |
|---|---|---|---|---|---|---|---|
| rs648997 | T | C | 0.026539 | 0.2581 | 3.2E-08 | 0.00479752 | 30.60099311 |
| rs6519133 | C | T | 0.0212412 | 0.396617 | 5.1E-07 | 0.00422785 | 25.24172059 |
| rs6734238 | G | A | -0.0246448 | 0.40289 | 4.90004E-09 | 0.00421347 | 34.21139505 |
| rs6855246 | G | A | -0.0406328 | 0.080147 | 2.59998E-07 | 0.0078869 | 26.54243927 |
| rs6935537 | C | T | -0.0279125 | 0.199177 | 9.20005E-08 | 0.00522502 | 28.53787017 |
| rs6939158 | C | G | -0.0524585 | 0.043297 | 1.09999E-06 | 0.0107551 | 23.79045953 |
| rs72623855 | A | G | -0.0489824 | 0.041035 | 3.09999E-06 | 0.0105023 | 21.75260539 |
| rs72842819 | C | A | 0.0343364 | 0.121254 | 7.19996E-08 | 0.00637379 | 29.02110993 |
| rs73225023 | T | C | -0.0377307 | 0.084605 | 3.89996E-07 | 0.00743304 | 25.7665804 |
| rs74892229 | A | G | -0.0514658 | 0.101705 | 4.60045E-14 | 0.00682197 | 56.91383434 |
| rs7599 | G | A | -0.0222618 | 0.631462 | 2E-07 | 0.00427861 | 27.07167638 |
| rs77542162 | G | A | -0.156008 | 0.022666 | 2.80027E-29 | 0.0138898 | 126.1542116 |
| rs77849807 | G | A | 0.080116 | 0.015244 | 2E-06 | 0.0168703 | 22.55240721 |
| rs79687284 | C | G | 0.0566294 | 0.034649 | 5.60003E-07 | 0.0113137 | 25.05385752 |
| rs8107347 | A | G | -0.0258045 | 0.349933 | 2.80001E-09 | 0.00434366 | 35.2922421 |
| rs819109 | A | G | 0.0196759 | 0.590467 | 3.69999E-06 | 0.00425213 | 21.41194632 |
| rs9389272 | A | G | 0.0271045 | 0.194236 | 2.5E-07 | 0.00525691 | 26.58408499 |
| rs9855653 | C | T | -0.0220889 | 0.589298 | 1.5E-07 | 0.00420644 | 27.57520698 |
| rs9912287 | A | G | 0.0415659 | 0.219307 | 1.20005E-16 | 0.00501919 | 68.58151974 |
| **11.Vitamin C-Keratoconus** | | | | | | | |
| rs114598078 | T | C | 0.0655782 | 0.042309 | 1.89998E-06 | 0.0137636 | 22.7008192753006 |
| rs11650824 | A | T | 0.0794787 | 0.035071 | 5.60003E-07 | 0.0158822 | 25.0418737145143 |

| | | | | | | | | |
|---|---|---|---|---|---|---|---|---|
| rs17482258 | T | C | 0.042829 | 0.099073 | 3.69999E-06 | 0.00926045 | 21.389386041938 |
| rs1883993 | A | G | 0.044959 | 0.095419 | 1.5E-06 | 0.00935324 | 23.1044567625811 |
| rs2018201 | G | T | -0.0808079 | 0.026551 | 2.5E-06 | 0.0171646 | 22.1629159539435 |
| rs4238567 | C | T | 0.0253068 | 0.522046 | 4.30002E-06 | 0.00550812 | 21.1083512234157 |
| rs4481190 | C | A | -0.0306375 | 0.351041 | 9.59997E-08 | 0.00574377 | 28.4511089788469 |
| rs61868302 | T | C | -0.0571013 | 0.060676 | 1.40001E-06 | 0.0118391 | 23.2616890655426 |
| rs74978963 | T | C | 0.150814 | 0.008729 | 1.2E-06 | 0.0310213 | 23.6346928508798 |
| rs7626478 | A | G | 0.0279796 | 0.720257 | 4.49997E-06 | 0.00610134 | 21.0290271470236 |
| rs9540734 | A | G | -0.0259285 | 0.477524 | 2.30001E-06 | 0.00548469 | 22.3479271388409 |

**12.Vitamin E-Keratoconus**

| | | | | | | | |
|---|---|---|---|---|---|---|---|
| rs111306778 | A | G | -0.0479658 | 0.089621 | 5.39995E-07 | 0.00957227 | 25.1084634973416 |
| rs12421920 | G | A | -0.0434479 | 0.093546 | 3.69999E-06 | 0.00938496 | 21.4318352002809 |
| rs12899673 | A | G | 0.0268951 | 0.333079 | 3.79997E-06 | 0.00581803 | 21.3688448938583 |
| rs2723979 | G | T | -0.0266142 | 0.583758 | 1.5E-06 | 0.00552745 | 23.1826895518784 |
| rs35218694 | G | A | -0.0741637 | 0.034459 | 1.29999E-06 | 0.0153104 | 23.4636912361338 |
| rs4903544 | T | C | -0.0295098 | 0.30035 | 9.49992E-07 | 0.0060211 | 24.0196947815733 |
| rs536912 | A | C | 0.0304596 | 0.736049 | 8.99995E-07 | 0.00620091 | 24.1281579999791 |
| rs6033 | G | A | -0.0516563 | 0.072493 | 9.90011E-07 | 0.0105563 | 23.9447207140033 |
| rs71385328 | G | A | 0.130015 | 0.011307 | 7.00003E-07 | 0.0262064 | 24.6126752840475 |
| rs79966958 | T | C | -0.116527 | 0.012689 | 2E-06 | 0.024505 | 22.6115508495519 |
| rs979218 | C | A | -0.0430325 | 0.098036 | 3.09999E-06 | 0.00922259 | 21.770779707939 |

SNP = single nucleotide polymorphisms, EA = effect allele, OA = other allele, Beta was obtained by allele-related effects; EAF = effect allele frequency, SE = standard error, F = F-statistics, SOD = super oxide dismutase, GST = glutathione transferase, GPX = glutathione peroxidase, CAT = catalase, MPO = Myeloperoxidase, PON = Paraoxonase, UA = uric acid, TBIL = total bilirubin.

**Supplementary Table S2. Characteristics of SNPs associated with Keratoconus.**

| SNPs | EA | OA | Beta | EAF | Pval | SE | F |
|---|---|---|---|---|---|---|---|
| **1.Keratoconus-SOD** | | | | | | | |
| rs118109996 | T | C | 2.0708 | 0.01221 | 4.43098E-06 | 0.4512 | 21.0638683 |
| rs142342363 | G | A | 7.6654 | 0.001627 | 2.119E-06 | 1.6166 | 22.48352988 |
| rs17598303 | T | C | 0.5747 | 0.1638 | 4.561E-07 | 0.1139 | 25.45862512 |
| rs2064656 | G | A | -0.3975 | 0.4848 | 9.13903E-07 | 0.081 | 24.08264746 |
| rs2683298 | A | G | 0.4038 | 0.4082 | 1.21099E-06 | 0.0832 | 23.55515556 |
| rs56037182 | A | C | 0.461 | 0.2135 | 4.54904E-06 | 0.1006 | 20.9993518 |
| rs75958235 | T | C | 5.2353 | 0.00277 | 3.16097E-06 | 1.1234 | 21.71772389 |
| rs7611197 | T | C | 0.4568 | 0.2233 | 3.43598E-06 | 0.0984 | 21.55073039 |
| **2.Keratoconus-GST** | | | | | | | |
| rs118109996 | T | C | 2.0708 | 0.01221 | 4.43098E-06 | 0.4512 | 21.0638683 |
| rs142342363 | G | A | 7.6654 | 0.001627 | 2.119E-06 | 1.6166 | 22.48352988 |
| rs17598303 | T | C | 0.5747 | 0.1638 | 4.561E-07 | 0.1139 | 25.45862512 |
| rs2064656 | G | A | -0.3975 | 0.4848 | 9.13903E-07 | 0.081 | 24.08264746 |
| rs2683298 | A | G | 0.4038 | 0.4082 | 1.21099E-06 | 0.0832 | 23.55515556 |
| rs56037182 | A | C | 0.461 | 0.2135 | 4.54904E-06 | 0.1006 | 20.9993518 |
| rs75958235 | T | C | 5.2353 | 0.00277 | 3.16097E-06 | 1.1234 | 21.71772389 |
| rs7611197 | T | C | 0.4568 | 0.2233 | 3.43598E-06 | 0.0984 | 21.55073039 |
| **3.Keratoconus-GPX** | | | | | | | |
| rs118109996 | T | C | 2.0708 | 0.01221 | 4.43098E-06 | 0.4512 | 21.0638683 |

| | | | | | | | |
|---|---|---|---|---|---|---|---|
| rs142342363 | G | A | 7.6654 | 0.001627 | 2.119E-06 | 1.6166 | 22.48352988 |
| rs17598303 | T | C | 0.5747 | 0.1638 | 4.561E-07 | 0.1139 | 25.45862512 |
| rs2064656 | G | A | -0.3975 | 0.4848 | 9.13903E-07 | 0.081 | 24.08264746 |
| rs2683298 | A | G | 0.4038 | 0.4082 | 1.21099E-06 | 0.0832 | 23.55515556 |
| rs56037182 | A | C | 0.461 | 0.2135 | 4.54904E-06 | 0.1006 | 20.9993518 |
| rs75958235 | T | C | 5.2353 | 0.00277 | 3.16097E-06 | 1.1234 | 21.71772389 |
| rs7611197 | T | C | 0.4568 | 0.2233 | 3.43598E-06 | 0.0984 | 21.55073039 |
| **4.Keratoconus-CAT** | | | | | | | |
| rs118109996 | T | C | 2.0708 | 0.01221 | 4.43098E-06 | 0.4512 | 21.0638683 |
| rs142342363 | G | A | 7.6654 | 0.001627 | 2.119E-06 | 1.6166 | 22.48352988 |
| rs17598303 | T | C | 0.5747 | 0.1638 | 4.561E-07 | 0.1139 | 25.45862512 |
| rs2064656 | G | A | -0.3975 | 0.4848 | 9.13903E-07 | 0.081 | 24.08264746 |
| rs2683298 | A | G | 0.4038 | 0.4082 | 1.21099E-06 | 0.0832 | 23.55515556 |
| rs56037182 | A | C | 0.461 | 0.2135 | 4.54904E-06 | 0.1006 | 20.9993518 |
| rs75958235 | T | C | 5.2353 | 0.00277 | 3.16097E-06 | 1.1234 | 21.71772389 |
| rs7611197 | T | C | 0.4568 | 0.2233 | 3.43598E-06 | 0.0984 | 21.55073039 |
| **5.Keratoconus-MPO** | | | | | | | |
| rs118109996 | T | C | 2.0708 | 0.01221 | 4.43098E-06 | 0.4512 | 21.0638683 |
| rs142342363 | G | A | 7.6654 | 0.001627 | 2.119E-06 | 1.6166 | 22.48352988 |
| rs17598303 | T | C | 0.5747 | 0.1638 | 4.561E-07 | 0.1139 | 25.45862512 |
| rs2064656 | G | A | -0.3975 | 0.4848 | 9.13903E-07 | 0.081 | 24.08264746 |

| | | | | | | | |
|---|---|---|---|---|---|---|---|
| rs2683298 | A | G | 0.4038 | 0.4082 | 1.21099E-06 | 0.0832 | 23.55515556 |
| rs56037182 | A | C | 0.461 | 0.2135 | 4.54904E-06 | 0.1006 | 20.9993518 |
| rs75958235 | T | C | 5.2353 | 0.00277 | 3.16097E-06 | 1.1234 | 21.71772389 |
| rs7611197 | T | C | 0.4568 | 0.2233 | 3.43598E-06 | 0.0984 | 21.55073039 |

**6.Keratoconus-PON**

| | | | | | | | |
|---|---|---|---|---|---|---|---|
| rs118109996 | T | C | 2.0708 | 0.01221 | 4.43098E-06 | 0.4512 | 21.0638683 |
| rs142342363 | G | A | 7.6654 | 0.001627 | 2.119E-06 | 1.6166 | 22.48352988 |
| rs17598303 | T | C | 0.5747 | 0.1638 | 4.561E-07 | 0.1139 | 25.45862512 |
| rs2064656 | G | A | -0.3975 | 0.4848 | 9.13903E-07 | 0.081 | 24.08264746 |
| rs2683298 | A | G | 0.4038 | 0.4082 | 1.21099E-06 | 0.0832 | 23.55515556 |
| rs56037182 | A | C | 0.461 | 0.2135 | 4.54904E-06 | 0.1006 | 20.9993518 |
| rs75958235 | T | C | 5.2353 | 0.00277 | 3.16097E-06 | 1.1234 | 21.71772389 |
| rs7611197 | T | C | 0.4568 | 0.2233 | 3.43598E-06 | 0.0984 | 21.55073039 |

**7.Keratoconus-UA**

| | | | | | | | |
|---|---|---|---|---|---|---|---|
| rs118109996 | T | C | 2.0708 | 0.01221 | 4.43098E-06 | 0.4512 | 21.0638683 |
| rs142342363 | G | A | 7.6654 | 0.001627 | 2.119E-06 | 1.6166 | 22.48352988 |
| rs2064656 | G | A | -0.3975 | 0.4848 | 9.13903E-07 | 0.081 | 24.08264746 |
| rs2683298 | A | G | 0.4038 | 0.4082 | 1.21099E-06 | 0.0832 | 23.55515556 |
| rs56037182 | A | C | 0.461 | 0.2135 | 4.54904E-06 | 0.1006 | 20.9993518 |
| rs75958235 | T | C | 5.2353 | 0.00277 | 3.16097E-06 | 1.1234 | 21.71772389 |
| rs7611197 | T | C | 0.4568 | 0.2233 | 3.43598E-06 | 0.0984 | 21.55073039 |

**8.Keratoconus-TBIL**

| | | | | | | | |
|---|---|---|---|---|---|---|---|
| rs118109996 | T | C | 2.0708 | 0.01221 | 4.43098E-06 | 0.4512 | 21.0638683 |
| rs142342363 | G | A | 7.6654 | 0.001627 | 2.119E-06 | 1.6166 | 22.48352988 |
| rs2064656 | G | A | -0.3975 | 0.4848 | 9.13903E-07 | 0.081 | 24.08264746 |
| rs2683298 | A | G | 0.4038 | 0.4082 | 1.21099E-06 | 0.0832 | 23.55515556 |
| rs56037182 | A | C | 0.461 | 0.2135 | 4.54904E-06 | 0.1006 | 20.9993518 |
| rs75958235 | T | C | 5.2353 | 0.00277 | 3.16097E-06 | 1.1234 | 21.71772389 |
| rs7611197 | T | C | 0.4568 | 0.2233 | 3.43598E-06 | 0.0984 | 21.55073039 |

**9.Keratoconus-Retinol**

| | | | | | | | |
|---|---|---|---|---|---|---|---|
| rs118109996 | T | C | 2.0708 | 0.01221 | 4.43098E-06 | 0.4512 | 21.0638683 |
| rs142342363 | G | A | 7.6654 | 0.001627 | 2.119E-06 | 1.6166 | 22.48352988 |
| rs17598303 | T | C | 0.5747 | 0.1638 | 4.561E-07 | 0.1139 | 25.45862512 |
| rs2064656 | G | A | -0.3975 | 0.4848 | 9.13903E-07 | 0.081 | 24.08264746 |
| rs2683298 | A | G | 0.4038 | 0.4082 | 1.21099E-06 | 0.0832 | 23.55515556 |
| rs56037182 | A | C | 0.461 | 0.2135 | 4.54904E-06 | 0.1006 | 20.9993518 |
| rs75958235 | T | C | 5.2353 | 0.00277 | 3.16097E-06 | 1.1234 | 21.71772389 |
| rs7611197 | T | C | 0.4568 | 0.2233 | 3.43598E-06 | 0.0984 | 21.55073039 |

**10.Keratoconus-Albumin**

| | | | | | | | |
|---|---|---|---|---|---|---|---|
| rs118109996 | T | C | 2.0708 | 0.01221 | 4.43098E-06 | 0.4512 | 21.0638683 |
| rs142342363 | G | A | 7.6654 | 0.001627 | 2.119E-06 | 1.6166 | 22.48352988 |
| rs17598303 | T | C | 0.5747 | 0.1638 | 4.561E-07 | 0.1139 | 25.45862512 |
| rs2064656 | G | A | -0.3975 | 0.4848 | 9.13903E-07 | 0.081 | 24.08264746 |

| | | | | | | | |
|---|---|---|---|---|---|---|---|
| rs2683298 | A | G | 0.4038 | 0.4082 | 1.21099E-06 | 0.0832 | 23.55515556 |
| rs56037182 | A | C | 0.461 | 0.2135 | 4.54904E-06 | 0.1006 | 20.9993518 |
| rs75958235 | T | C | 5.2353 | 0.00277 | 3.16097E-06 | 1.1234 | 21.71772389 |
| rs7611197 | T | C | 0.4568 | 0.2233 | 3.43598E-06 | 0.0984 | 21.55073039 |

**11.Keratoconus-Vitamin C**

| | | | | | | | |
|---|---|---|---|---|---|---|---|
| rs118109996 | T | C | 2.0708 | 0.01221 | 4.43098E-06 | 0.4512 | 21.0638683 |
| rs142342363 | G | A | 7.6654 | 0.001627 | 2.119E-06 | 1.6166 | 22.48352988 |
| rs17598303 | T | C | 0.5747 | 0.1638 | 4.561E-07 | 0.1139 | 25.45862512 |
| rs2064656 | G | A | -0.3975 | 0.4848 | 9.13903E-07 | 0.081 | 24.08264746 |
| rs2683298 | A | G | 0.4038 | 0.4082 | 1.21099E-06 | 0.0832 | 23.55515556 |
| rs56037182 | A | C | 0.461 | 0.2135 | 4.54904E-06 | 0.1006 | 20.9993518 |
| rs75958235 | T | C | 5.2353 | 0.00277 | 3.16097E-06 | 1.1234 | 21.71772389 |
| rs7611197 | T | C | 0.4568 | 0.2233 | 3.43598E-06 | 0.0984 | 21.55073039 |

**12.Keratoconus-Vitamin E**

| | | | | | | | |
|---|---|---|---|---|---|---|---|
| rs118109996 | T | C | 2.0708 | 0.01221 | 4.43098E-06 | 0.4512 | 21.0638683 |
| rs142342363 | G | A | 7.6654 | 0.001627 | 2.119E-06 | 1.6166 | 22.48352988 |
| rs17598303 | T | C | 0.5747 | 0.1638 | 4.561E-07 | 0.1139 | 25.45862512 |
| rs2064656 | G | A | -0.3975 | 0.4848 | 9.13903E-07 | 0.081 | 24.08264746 |
| rs2683298 | A | G | 0.4038 | 0.4082 | 1.21099E-06 | 0.0832 | 23.55515556 |
| rs56037182 | A | C | 0.461 | 0.2135 | 4.54904E-06 | 0.1006 | 20.9993518 |

| | | | | | | | |
|---|---|---|---|---|---|---|---|
| rs75958235 | T | C | 5.2353 | 0.00277 | 3.16097E-06 | 1.1234 | 21.71772389 |
| rs7611197 | T | C | 0.4568 | 0.2233 | 3.43598E-06 | 0.0984 | 21.55073039 |

SNP = single nucleotide polymorphisms, EA = effect allele, OA = other allele, Beta was obtained by allele-related effects; EAF = effect allele frequency, SE = standard error, F = F-statistics, SOD = super oxide dismutase, GST = glutathione transferase, GPX = glutathione peroxidase, CAT = catalase, MPO = Myeloperoxidase, PON = Paraoxonase, UA = uric acid, TBIL = total bilirubin.

**Supplementary Table S3. Results of forward MR analysis under different methods.**

| Exposure | Outcome | nSNPs | Methods | OR | 95%CI-Low | 95%CI-Up | β | SE | *P* value |
|---|---|---|---|---|---|---|---|---|---|
| SOD | Keratoconus | 13 | MR Egger | 0.893 | 0.431 | 1.847 | -0.114 | 0.371 | 0.765 |
| | | | Weighted median | 1.191 | 0.769 | 1.845 | 0.175 | 0.223 | 0.433 |
| | | | Inverse variance weighted | 1.129 | 0.806 | 1.581 | 0.121 | 0.172 | 0.481 |
| | | | Simple mode | 1.230 | 0.627 | 2.414 | 0.207 | 0.344 | 0.558 |
| | | | Weighted mode | 0.952 | 0.530 | 1.713 | -0.049 | 0.299 | 0.873 |
| GST | | 11 | MR Egger | 1.281 | 0.769 | 2.133 | 0.248 | 0.260 | 0.366 |
| | | | Weighted median | 1.120 | 0.815 | 1.539 | 0.113 | 0.162 | 0.486 |
| | | | Inverse variance weighted | 1.051 | 0.818 | 1.350 | 0.050 | 0.128 | 0.697 |
| | | | Simple mode | 1.096 | 0.686 | 1.753 | 0.092 | 0.239 | 0.709 |
| | | | Weighted mode | 1.124 | 0.818 | 1.544 | 0.116 | 0.162 | 0.489 |
| GPX | | 14 | MR Egger | 0.747 | 0.443 | 1.258 | -0.292 | 0.266 | 0.294 |
| | | | Weighted median | 0.847 | 0.593 | 1.211 | -0.166 | 0.182 | 0.363 |
| | | | Inverse variance weighted | 0.660 | 0.500 | 0.873 | -0.415 | 0.142 | 0.004 |
| | | | Simple mode | 0.454 | 0.184 | 1.122 | -0.790 | 0.461 | 0.111 |
| | | | Weighted mode | 0.907 | 0.621 | 1.324 | -0.098 | 0.193 | 0.621 |
| CAT | | 14 | MR Egger | 0.467 | 0.177 | 1.232 | -0.761 | 0.495 | 0.150 |
| | | | Weighted median | 0.916 | 0.576 | 1.457 | -0.087 | 0.237 | 0.712 |
| | | | Inverse variance weighted | 1.136 | 0.819 | 1.576 | 0.128 | 0.167 | 0.444 |
| | | | Simple mode | 0.929 | 0.453 | 1.909 | -0.073 | 0.367 | 0.845 |
| | | | Weighted mode | 0.878 | 0.471 | 1.637 | -0.130 | 0.318 | 0.690 |
| MPO | | 29 | MR Egger | 0.997 | 0.508 | 1.956 | -0.003 | 0.344 | 0.992 |
| | | | Weighted median | 1.238 | 0.703 | 2.182 | 0.214 | 0.289 | 0.460 |
| | | | Inverse variance weighted | 1.181 | 0.783 | 1.782 | 0.167 | 0.210 | 0.427 |
| | | | Simple mode | 0.867 | 0.333 | 2.260 | -0.142 | 0.489 | 0.773 |
| | | | Weighted mode | 1.245 | 0.695 | 2.230 | 0.219 | 0.297 | 0.467 |
| PON | | 19 | MR Egger | 0.939 | 0.634 | 1.392 | -0.063 | 0.201 | 0.759 |
| | | | Weighted median | 1.042 | 0.803 | 1.351 | 0.041 | 0.133 | 0.759 |
| | | | Inverse variance weighted | 1.079 | 0.888 | 1.310 | 0.076 | 0.099 | 0.444 |
| | | | Simple mode | 0.808 | 0.507 | 1.289 | -0.213 | 0.238 | 0.384 |
| | | | Weighted mode | 0.914 | 0.556 | 1.503 | -0.090 | 0.254 | 0.726 |
| UA | | 372 | MR Egger | 1.002 | 0.992 | 1.013 | 0.002 | 0.005 | 0.640 |
| | | | Weighted median | 1.007 | 0.997 | 1.017 | 0.007 | 0.005 | 0.182 |

| | | | | | | | | |
|---|---|---|---|---|---|---|---|---|
| | | Inverse variance weighted | 1.004 | 0.998 | 1.010 | 0.004 | 0.003 | 0.237 |
| | | Simple mode | 1.021 | 0.999 | 1.043 | 0.021 | 0.011 | 0.060 |
| | | Weighted mode | 1.006 | 0.996 | 1.016 | 0.006 | 0.005 | 0.230 |
| TBIL | 144 | MR Egger | 0.910 | 0.835 | 0.992 | -0.094 | 0.044 | 0.034 |
| | | Weighted median | 0.903 | 0.810 | 1.007 | -0.102 | 0.056 | 0.067 |
| | | Inverse variance weighted | 0.912 | 0.845 | 0.984 | -0.093 | 0.039 | 0.017 |
| | | Simple mode | 0.963 | 0.677 | 1.371 | -0.037 | 0.180 | 0.836 |
| | | Weighted mode | 0.923 | 0.844 | 1.008 | -0.080 | 0.045 | 0.078 |
| Retinol | 8 | MR Egger | 0.580 | 0.005 | 65.221 | -0.544 | 2.409 | 0.829 |
| | | Weighted median | 3.305 | 0.213 | 51.327 | 1.195 | 1.399 | 0.393 |
| | | Inverse variance weighted | 3.496 | 0.489 | 25.004 | 1.252 | 1.004 | 0.212 |
| | | Simple mode | 22.797 | 0.449 | 1157.575 | 3.127 | 2.004 | 0.163 |
| | | Weighted mode | 3.043 | 0.137 | 67.543 | 1.113 | 1.582 | 0.504 |
| Albumin | 77 | MR Egger | 0.833 | 0.265 | 2.618 | -0.183 | 0.584 | 0.755 |
| | | Weighted median | 0.955 | 0.310 | 2.935 | -0.046 | 0.573 | 0.935 |
| | | Inverse variance weighted | 0.756 | 0.392 | 1.459 | -0.280 | 0.335 | 0.404 |
| | | Simple mode | 1.142 | 0.125 | 10.455 | 0.133 | 1.130 | 0.907 |
| | | Weighted mode | 0.908 | 0.261 | 3.161 | -0.096 | 0.636 | 0.880 |
| Vitamin C | 11 | MR Egger | 0.330 | 0.014 | 8.012 | -1.109 | 1.628 | 0.513 |
| | | Weighted median | 2.247 | 0.263 | 19.198 | 0.810 | 1.094 | 0.459 |
| | | Inverse variance weighted | 1.489 | 0.307 | 7.224 | 0.398 | 0.806 | 0.621 |
| | | Simple mode | 3.500 | 0.124 | 98.623 | 1.253 | 1.703 | 0.479 |
| | | Weighted mode | 3.275 | 0.203 | 52.914 | 1.186 | 1.420 | 0.423 |
| Vitamin E | 11 | MR Egger | 0.074 | 0.002 | 2.636 | -2.604 | 1.823 | 0.187 |
| | | Weighted median | 0.303 | 0.037 | 2.517 | -1.193 | 1.079 | 0.269 |
| | | Inverse variance weighted | 0.494 | 0.077 | 3.153 | -0.705 | 0.946 | 0.456 |
| | | Simple mode | 0.406 | 0.015 | 11.010 | -0.900 | 1.683 | 0.604 |
| | | Weighted mode | 0.305 | 0.031 | 2.978 | -1.189 | 1.163 | 0.331 |

SOD = super oxide dismutase, GST = glutathione transferase, GPX = glutathione peroxidase, CAT = catalase, MPO = Myeloperoxidase, PON = Paraoxonase, UA = uric acid, TBIL = total bilirubin, MR = Mendelian randomization, SNPs = single-nucleotide polymorphisms, OR = odds ratio, CI = confidence interval, SE = standard error.

**Supplementary Table S4. Results of reverse MR analysis under different methods.**

| Exposure | Outcome | nSNPs | Methods | OR | 95%CI-Low | 95%CI-Up | β | SE | *P* value |
|---|---|---|---|---|---|---|---|---|---|
| Keratoconus | SOD | 8 | MR Egger | 1.006 | 0.982 | 1.030 | 0.006 | 0.012 | 0.658 |
| | | | Weighted median | 1.006 | 0.982 | 1.030 | 0.006 | 0.012 | 0.636 |
| | | | Inverse variance weighted | 1.001 | 0.981 | 1.021 | 0.001 | 0.010 | 0.919 |
| | | | Simple mode | 1.008 | 0.972 | 1.047 | 0.008 | 0.019 | 0.669 |
| | | | Weighted mode | 1.006 | 0.982 | 1.030 | 0.006 | 0.012 | 0.662 |
| | GST | 8 | MR Egger | 0.996 | 0.973 | 1.020 | -0.004 | 0.012 | 0.775 |
| | | | Weighted median | 0.998 | 0.974 | 1.023 | -0.002 | 0.013 | 0.863 |
| | | | Inverse variance weighted | 0.997 | 0.977 | 1.017 | -0.003 | 0.010 | 0.743 |
| | | | Simple mode | 0.995 | 0.958 | 1.032 | -0.005 | 0.019 | 0.790 |
| | | | Weighted mode | 0.998 | 0.976 | 1.020 | -0.002 | 0.011 | 0.862 |
| | GPX | 8 | MR Egger | 0.991 | 0.968 | 1.015 | -0.009 | 0.012 | 0.476 |
| | | | Weighted median | 0.988 | 0.963 | 1.014 | -0.012 | 0.013 | 0.363 |
| | | | Inverse variance weighted | 0.995 | 0.975 | 1.015 | -0.005 | 0.010 | 0.610 |
| | | | Simple mode | 1.000 | 0.955 | 1.048 | 0.000 | 0.024 | 0.984 |
| | | | Weighted mode | 0.986 | 0.961 | 1.011 | -0.015 | 0.013 | 0.296 |
| | CAT | 8 | MR Egger | 0.975 | 0.947 | 1.003 | -0.026 | 0.014 | 0.126 |
| | | | Weighted median | 0.984 | 0.960 | 1.009 | -0.016 | 0.013 | 0.209 |
| | | | Inverse variance weighted | 0.987 | 0.961 | 1.014 | -0.013 | 0.014 | 0.340 |
| | | | Simple mode | 0.979 | 0.938 | 1.021 | -0.021 | 0.022 | 0.356 |
| | | | Weighted mode | 0.983 | 0.961 | 1.006 | -0.017 | 0.012 | 0.189 |

| | | | | | | | | |
|---|---|---|---|---|---|---|---|---|
| MPO | 8 | MR Egger | 1.003 | 0.987 | 1.019 | 0.003 | 0.008 | 0.697 |
| | | Weighted median | 1.002 | 0.989 | 1.015 | 0.002 | 0.007 | 0.724 |
| | | Inverse variance weighted | 1.004 | 0.992 | 1.016 | 0.004 | 0.006 | 0.561 |
| | | Simple mode | 1.007 | 0.984 | 1.030 | 0.006 | 0.012 | 0.596 |
| | | Weighted mode | 1.003 | 0.990 | 1.016 | 0.003 | 0.007 | 0.667 |
| PON | 8 | MR Egger | 1.002 | 0.966 | 1.038 | 0.002 | 0.018 | 0.930 |
| | | Weighted median | 1.008 | 0.978 | 1.038 | 0.008 | 0.015 | 0.617 |
| | | Inverse variance weighted | 1.013 | 0.981 | 1.046 | 0.013 | 0.016 | 0.436 |
| | | Simple mode | 0.974 | 0.908 | 1.046 | -0.026 | 0.036 | 0.494 |
| | | Weighted mode | 1.007 | 0.980 | 1.034 | 0.007 | 0.014 | 0.646 |
| UA | 7 | MR Egger | 1.209 | 1.023 | 1.429 | 0.190 | 0.085 | 0.076 |
| | | Weighted median | 1.166 | 0.970 | 1.401 | 0.153 | 0.094 | 0.103 |
| | | Inverse variance weighted | 1.121 | 0.960 | 1.309 | 0.114 | 0.079 | 0.149 |
| | | Simple mode | 0.995 | 0.714 | 1.386 | -0.005 | 0.169 | 0.976 |
| | | Weighted mode | 1.222 | 1.015 | 1.470 | 0.200 | 0.094 | 0.078 |
| TBIL | 7 | MR Egger | 0.998 | 0.988 | 1.009 | -0.002 | 0.005 | 0.766 |
| | | Weighted median | 0.997 | 0.987 | 1.008 | -0.003 | 0.005 | 0.591 |
| | | Inverse variance weighted | 0.997 | 0.988 | 1.006 | -0.003 | 0.005 | 0.515 |
| | | Simple mode | 0.983 | 0.965 | 1.001 | -0.018 | 0.009 | 0.113 |
| | | Weighted mode | 1.000 | 0.989 | 1.010 | 0.000 | 0.005 | 0.951 |
| Retinol | 8 | MR Egger | 0.997 | 0.989 | 1.005 | -0.003 | 0.004 | 0.529 |
| | | Weighted median | 0.998 | 0.992 | 1.004 | -0.002 | 0.003 | 0.524 |

| | | | | | | | | |
|---|---|---|---|---|---|---|---|---|
| | | Inverse variance weighted | 0.997 | 0.991 | 1.003 | -0.003 | 0.003 | 0.383 |
| | | Simple mode | 0.987 | 0.977 | 0.997 | -0.013 | 0.005 | 0.034 |
| | | Weighted mode | 0.998 | 0.992 | 1.004 | -0.002 | 0.003 | 0.547 |
| Albumin | 8 | MR Egger | 1.004 | 1.000 | 1.008 | 0.004 | 0.002 | 0.134 |
| | | Weighted median | 1.003 | 0.998 | 1.007 | 0.003 | 0.002 | 0.202 |
| | | Inverse variance weighted | 1.002 | 0.998 | 1.006 | 0.002 | 0.002 | 0.267 |
| | | Simple mode | 1.002 | 0.995 | 1.009 | 0.002 | 0.004 | 0.588 |
| | | Weighted mode | 1.003 | 0.999 | 1.007 | 0.003 | 0.002 | 0.207 |
| Vitamin C | 8 | MR Egger | 0.999 | 0.992 | 1.007 | -0.001 | 0.004 | 0.879 |
| | | Weighted median | 0.999 | 0.992 | 1.005 | -0.001 | 0.003 | 0.659 |
| | | Inverse variance weighted | 0.999 | 0.993 | 1.004 | -0.001 | 0.003 | 0.686 |
| | | Simple mode | 1.008 | 0.995 | 1.021 | 0.008 | 0.006 | 0.252 |
| | | Weighted mode | 0.999 | 0.993 | 1.004 | -0.001 | 0.003 | 0.644 |
| Vitamin E | 8 | MR Egger | 0.995 | 0.988 | 1.002 | -0.005 | 0.004 | 0.227 |
| | | Weighted median | 0.994 | 0.987 | 1.001 | -0.006 | 0.003 | 0.074 |
| | | Inverse variance weighted | 0.995 | 0.990 | 1.001 | -0.005 | 0.003 | 0.089 |
| | | Simple mode | 0.996 | 0.985 | 1.007 | -0.004 | 0.005 | 0.481 |
| | | Weighted mode | 0.994 | 0.987 | 1.000 | -0.007 | 0.003 | 0.089 |

SOD = super oxide dismutase, GST = glutathione transferase, GPX = glutathione peroxidase, CAT = catalase, MPO = Myeloperoxidase, PON = Paraoxonase, UA = uric acid, TBIL = total bilirubin, MR = Mendelian randomization, SNPs = single-nucleotide polymorphisms, OR = odds ratio, CI = confidence interval, SE = standard error.

**Supplementary Table S5. Pleiotropy and Heterogeneity Test of the reverse MR analysis.**

| | | | Horizontal pleiotropy test | | | Heterogeneity test (IVW) | | | | MR PRESSO |
|---|---|---|---|---|---|---|---|---|---|---|
| | | | MR-Egger | | | IVW | | MR-Egger | | |
| **Exposure** | **Outcome** | **nSNPs** | **Intercept** | **SE** | **p_intercept** | **Q** | **Q_pval** | **Q** | **Q_pval** | **Global test *P*** |
| Keratoconus | SOD | 8 | -1.05E-02 | 0.015 | 0.505 | 3.425 | 0.843 | 2.924 | 0.818 | 0.869 |
| | GST | 8 | 6.23E-04 | 0.015 | 0.968 | 2.480 | 0.929 | 2.478 | 0.871 | 0.967 |
| | GPX | 8 | 9.15E-03 | 0.015 | 0.561 | 3.921 | 0.789 | 3.542 | 0.738 | 0.617 |
| | CAT | 8 | 2.93E-02 | 0.018 | 0.152 | 12.572 | 0.083 | 8.686 | 0.192 | 0.209 |
| | MPO | 8 | 3.77E-04 | 0.008 | 0.964 | 9.440 | 0.223 | 9.437 | 0.150 | 0.371 |
| | PON | 8 | 3.43E-02 | 0.028 | 0.273 | 10.810 | 0.147 | 8.693 | 0.191 | 0.331 |
| | UA | 7 | -1.81E-01 | 0.106 | 0.150 | 7.104 | 0.311 | 4.212 | 0.519 | 0.373 |
| | TBIL | 7 | -3.10E-03 | 0.007 | 0.663 | 4.373 | 0.626 | 4.159 | 0.527 | 0.612 |
| | Retinol | 8 | -2.55E-05 | 0.005 | 0.996 | 11.996 | 0.101 | 11.996 | 0.062 | 0.229 |
| | Albumin | 8 | -3.60E-03 | 0.002 | 0.195 | 6.439 | 0.490 | 4.311 | 0.635 | 0.612 |
| | Vitamin C | 8 | -1.16E-03 | 0.004 | 0.790 | 9.719 | 0.205 | 9.594 | 0.143 | 0.336 |
| | Vitamin E | 8 | 2.58E-04 | 0.004 | 0.953 | 9.796 | 0.200 | 9.789 | 0.134 | 0.294 |

SOD = super oxide dismutase, GST = glutathione transferase, GPX = glutathione peroxidase, CAT = catalase, MPO = Myeloperoxidase, PON = Paraoxonase, UA = uric acid, TBIL = total bilirubin, SNPs = single-nucleotide polymorphisms, SE = standard error, IVW = inverse variance weighted, MR = Mendelian randomization, MR-PRESSO = MR pleiotropy residual sum and outlier.
